# Conformable Microelectrode Arrays Integrated with a Scoop‐Shaped Slide‐Well for Dynamic Electrophysiological Profiling of Patient‐Derived Cardiac Organoids

**DOI:** 10.1002/advs.202514365

**Published:** 2026-03-30

**Authors:** Ye Seul Kim, Jeonghwa Jeong, Gyeonghwa Heo, Young Woo Kwon, Jeongha Lee, Yoon Ji Jeong, Gyu Tae Park, Seongdo Jeong, Ki Won Hwang, Murim Choi, Jinhong Shin, Suck Won Hong, Jae Ho Kim

**Affiliations:** ^1^ Department of Physiology Department of Convergence Stem Cell Research Center School of Medicine Pusan National University Yangsan Republic of Korea; ^2^ Optics and Mechatronics Engineering Major Department of Cogno‐Mechatronics Engineering School of Transdisciplinary Engineering Pusan National University Busan Republic of Korea; ^3^ Engineering Research Center for Color‐Modulated Extra‐Sensory Perception Technology Pusan National University Busan Republic of Korea; ^4^ Department of Biomedical Sciences College of Medicine Seoul National University Seoul Republic of Korea; ^5^ Research Institute for Convergence of Biomedical Science and Technology Pusan National University Yangsan Hospital Yangsan Republic of Korea; ^6^ Division of Cardiology Department of Internal Medicine School of Medicine Pusan National University Yangsan Hospital Yangsan Republic of Korea; ^7^ Department of Neurology College of Medicine Pusan National University Yangsan Hospital Yangsan Republic of Korea

**Keywords:** cardiac organoid, cardiomyopathy, duchenne muscular dystrophy, electrophysiology, microelectrode array

## Abstract

We present an integrated microelectrode array (MEA) platform for high‐precision electrophysiological characterization of patient‐derived 3D cardiac organoids (COs), enabling dynamic recordings from both healthy and Duchenne muscular dystrophy (DMD) “mini‐hearts.” A deformable MEA, fabricated by photolithographic patterning of support layers and microelectrodes, is conformally assembled onto a customized scoop‐slide well. The concave geometry enables passive self‐alignment of millimeter‐scale COs within a multichannel electrode array, allowing simultaneous extracellular field potential (EFP) recording and video‐based motion analysis. Using COs derived from human induced pluripotent stem cells of healthy donors and a DMD patient, we resolved region‐specific EFP waveforms, which were validated by calcium imaging. DMD COs exhibited severe arrhythmic firing and aberrant waveform morphologies, in contrast to the stable, isochronal rhythms of healthy controls. Single‐cell transcriptomic analysis revealed a pathological DMD signature marked by excessive extracellular matrix accumulation and disorganized cardiac architecture, contributing to conduction heterogeneity. Pharmacological challenge with the hERG blocker E‐4031 prolonged field potential duration in normal COs but triggered disorganized arrhythmogenic storms in DMD COs. By overcoming the limitations of rigid interfaces and ensuring structural preservation with stable impedance coupling, the presented platform provides a robust biointerface for disease phenotyping, drug screening, and mechanistic interrogation of 3D cardiac tissues.

## Graphical abstract

A flexible, ultrathin multi‐channel microelectrode array (MEA) conformally integrated onto a curved slide‐well enables stable electrical interfacing with 3D cardiac organoids. The geometry‐guided self‐alignment allows simultaneous recording of extracellular field potentials and contractile motion from irregular, beating tissues. This platform provides a robust bioelectronic interface for functional phenotyping and drug response analysis in patient‐derived cardiac organoids.

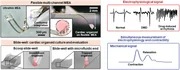

## 1 Introduction

Duchenne muscular dystrophy (DMD), one of the most severe forms of inherited muscular dystrophy, is caused by a mutation in the DMD (Xp21.2‐p21.1 [MIM: 300377]) gene, which affects approximately 1 in 5000 male births. DMD is characterized by progressive muscle weakness and degeneration, affecting both skeletal and cardiac muscles. Patients commonly experience cardiac and orthopedic complications, often leading to premature death in their 20s due to respiratory muscle weakness or cardiomyopathy [1]. Despite extensive research efforts, there is currently no known cure for DMD, and treatment options are limited to delaying the onset of symptoms [2]. Specifically, the primary cardiac manifestation associated with DMD is dilated cardiomyopathy, triggered by sarcolemmal fragility and downstream signaling cascades including inflammation and ion channel dysregulation [3]. The hearts of DMD patients consequently become overloaded, with dilated left ventricles, decreased contractility, and eventually congestive heart failure [4]. The development of novel therapeutic interventions targeting DMD is an intricate and arduous process, further compounded by the substantial financial burden imposed on patients due to the high cost of these treatments [5]. Given the challenges in conducting clinical studies in humans, there is an urgent need to develop disease models that faithfully recapitulate the structural and functional characteristics of the DMD patient's heart.

Organoids, 3D cellular structures that autonomously organize into organ‐like structures in vitro, have emerged as a revolutionary platform in biomedical research [6]. These self‐organizing 3D cell cultures have made remarkable strides in recapitulating the anatomy, cellular diversity, and functional features of human organs to model diseases from neurological to muscular disorders. Cardiac organoids (CO) can be fabricated from human induced pluripotent stem cells (hiPSCs) and include cardiomyocytes (CMs), endothelial cells, and smooth muscle cells, which are essential for cardiac function [7]. Recent studies have reported the generation of self‐organizing CO with ventricles and major cardiac components, including the ventricular outflow tract and atrioventricular canal, serving as a novel platform to model congenital heart malformations [8, 9]. Functionally, CO can represent the cardiac system with a more complete electrical signaling mechanism than 2D CM cultures. In addition, co‐emergence of the gut/intestine, an endoderm‐derived lineage, in close proximity to the myocardium stimulated CM maturation [10], suggesting that organoid models are useful for examination of multi‐tissue interactions during development, physiological maturation, and diseases. Recently, it has been reported that CO generated from DMD patient‐derived iPSCs exhibited CM deterioration, fibrosis, and displayed DMD‐related cardiomyopathy phenotypes [11]. Therefore, CO is highly versatile for modeling cardiac dysfunctions associated with DMD. Importantly, the morphogenetic progression of cardiac organoids introduces substantial geometric and electrophysiological heterogeneity that fundamentally challenges conventional planar bioelectronic interfaces. Unlike monolayer cardiomyocyte cultures, organoids exhibit curvature, regional subtype diversification, endogenous extracellular matrix remodeling, and evolving conduction anisotropy, all of which are features that directly compromise electrode–tissue conformity, spatial resolution, and signal reproducibility in traditional 2D MEA platforms. Therefore, resolving organoid developmental complexity is not merely a biological objective but an engineering prerequisite for designing a stable, high‐fidelity electrophysiological interface.

In this context, drug development for cardiomyopathy patients is a meticulous process that requires extensive testing to assess the therapeutic potential and possible adverse effects of new compounds. A crucial aspect of this process involves analyzing electrophysiological properties and evaluating the risk of drug‐induced arrhythmias, such as torsade de pointes (TdP), a potentially life‐threatening cardiac arrhythmia associated with QT interval prolongation. Traditionally, the assessment of arrhythmia risk for hiPSC‐derived CMs has been conducted using conventional 2D microelectrode arrays (MEAs). However, recent advancements have led to the development of CO as a novel and promising platform for drug screening [12]. These organoid models enabled researchers to evaluate the biological mechanisms underlying cardiac contraction, revealing significant differences compared to hiPSC‐derived CMs cultured on 2D MEAs. In particular, because CO exhibits dynamic responses similar to their in vivo counterparts, it is critical to monitor their activity through technological advancements. Recent reports have highlighted the development of MEAs, either embedded in well‐plates or integrated as flexible nets wrapped around organoids, as a promising approach for analyzing electrophysiological signals from 3D structures, such as the brain or CO [13, 14, 15]. Recently developed MEA systems with bioelectronic interfaces have been presented for 2D and 3D cultures, including planar MEAs for monolayer cardiac cultures and transformable electrode arrays for neural organoids. However, the currently reported device structures, while offering the advantage of high uniformity, are not optimally suited for measuring non‐uniformly shaped cultured CO [16]. Furthermore, these systems often lack MEAs optimized for culturing 3D CO, limiting their potential for comprehensive analysis.

**SCHEME 1:**
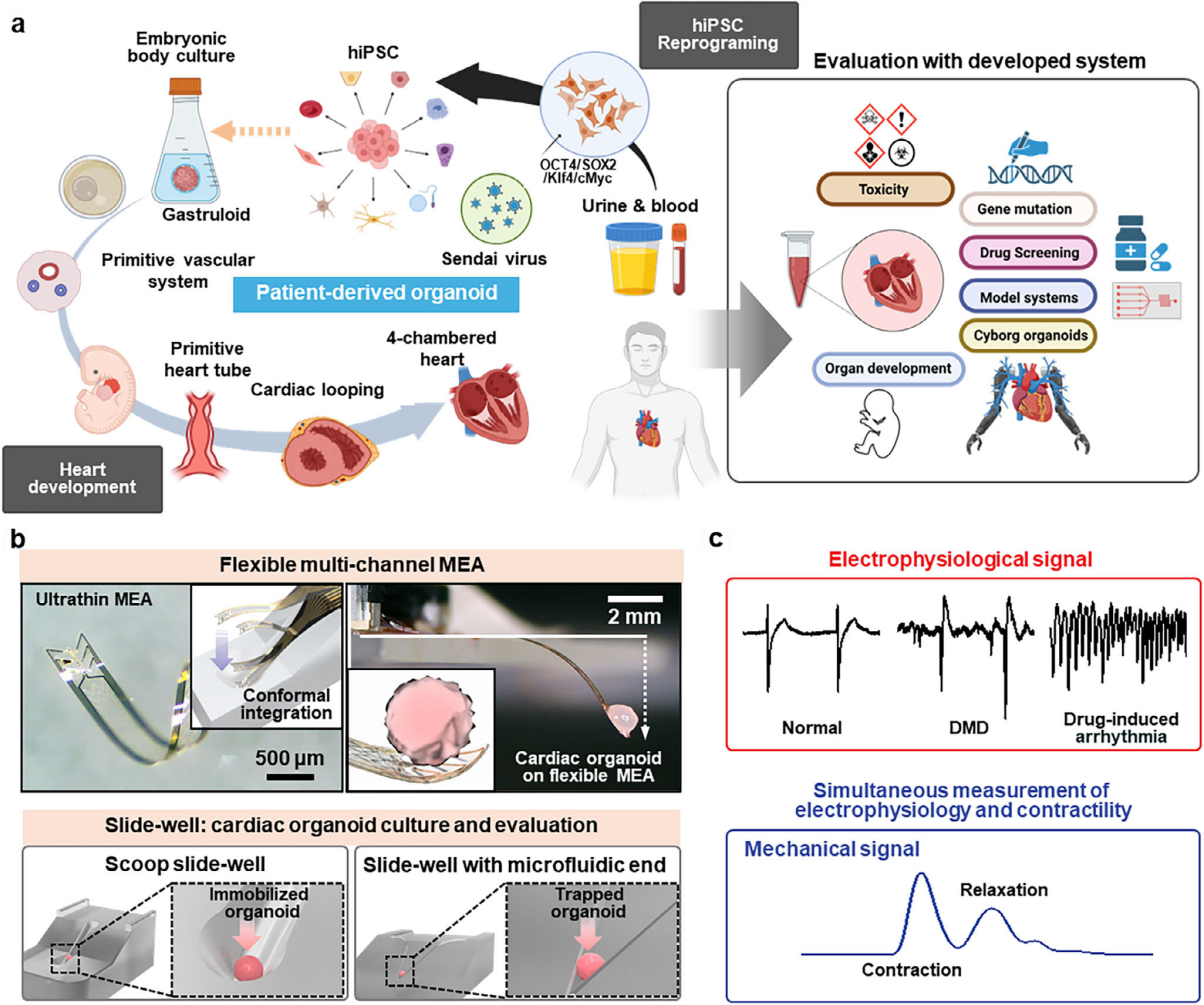
Engineering concept of the scoop‐integrated deformable MEA platform. (a) Generation of cardiac organoids from hiPSCs reprogrammed using patient urine‐derived somatic cells, followed by differentiation mimicking human heart development stages from primitive vascular structures to a chambered heart. Created in BioRender (Smith, J., 2025; BioRender.com/c248457) (b) Geometrically adaptive MEA integration within a scoop‐shaped slide‐well to ensure conformal contact with curved 3D tissues. (c) Functional validation through spatially resolved electrophysiological and mechanical phenotyping of normal and DMD organoids for disease discrimination and drug‐response profiling.

Herein, we report a strategically integrated platform for culturing CO derived from genetically diseased donors and interrogating their pathophysiological signatures via a bioengineered interface. To address the morphological complexity of CO, our geometrically tunable scoop‐shaped MEAs enable real‐time monitoring of cardiac functional metrics, including drug‐evoked contractile responses and associated perturbations in beating dynamics. Leveraging this integrated platform, we systematically compared structural, phenotypic, and electrophysiological divergences between diseased and healthy CO, with a focus on arrhythmogenic susceptibility following pharmacological challenge (e.g., E‐4031). Crucially, we validated the robust differentiation of hiPSCs, sourced from non‐invasively acquired blood and urine samples of healthy donors and genetic cardiomyopathy patients, into self‐organizing chamber‐like CO (“mini‐hearts,” Scheme 1a). These constructions enabled multipoint electrophysiological mapping across distinct anatomical regions of interest. A key challenge arose from the irregular morphology of CO, which compromises electrode‐tissue conformity in conventional 2D MEAs, precluding reliable electrophysiological interrogation. To circumvent this limitation, we engineered a 3D‐structured biointerface spatially optimized to resolve contractile behavior and electrochemical signaling in morphologically heterogeneous CO. Our approach overcomes intrinsic drawbacks of traditional MEAs, such as low spatial resolution, restricted signal mapping fidelity, and inability to resolve signals from morphologically complex organoids.

Emphasizing the technical innovations, we designed a flexible MEA incorporating ultrathin layered materials. This design enables the precise recording of electrophysiological signals from 3D CO with irregular topographies and millimeter‐scale sizes. Additionally, the deformable MEAs were synergistically integrated with a custom‐designed scoop‐shaped slide‐well, forming a bioelectronic interface capable of accommodating morphologically heterogeneous CO while holding culture media (Scheme 1b). With this engineered platform, electrophysiological signals were stably measured by providing a fluidic pathway that guided organoids to roll over and become trapped on the target MEA surface. The scoop‐shaped culture vessel promoted gentle electrode‐organoid interfacing, minimizing mechanical stress while maximizing signal fidelity. Our results demonstrated that the microstructured electrodes, conformally mounted within the scoop slide‐well, collected electrophysiological data from CO with high accuracy. The implications of these advances extend beyond currently reported methods, enabling a broader range of applications. Notably, as outlined in Scheme 1c, the platform supports patient‐specific disease modeling and pharmacological screening, particularly when coupled with personalized medicine. Studies using CO derived from hiPSCs represent a significant step forward in comprehensively analyzing and understanding complex organoid models for therapeutic discovery. Furthermore, the extension of this technological concept offers value‐added opportunities for acquiring electrophysiological data from bespoke biological samples, such as CO, with potential applications in various biomedical research fields.

## 2 Results

### 2.1 Design of Deformable MEA Integrated with Scoop Side‐Well for Recording Electrophysiological Signals

**FIGURE 1:**
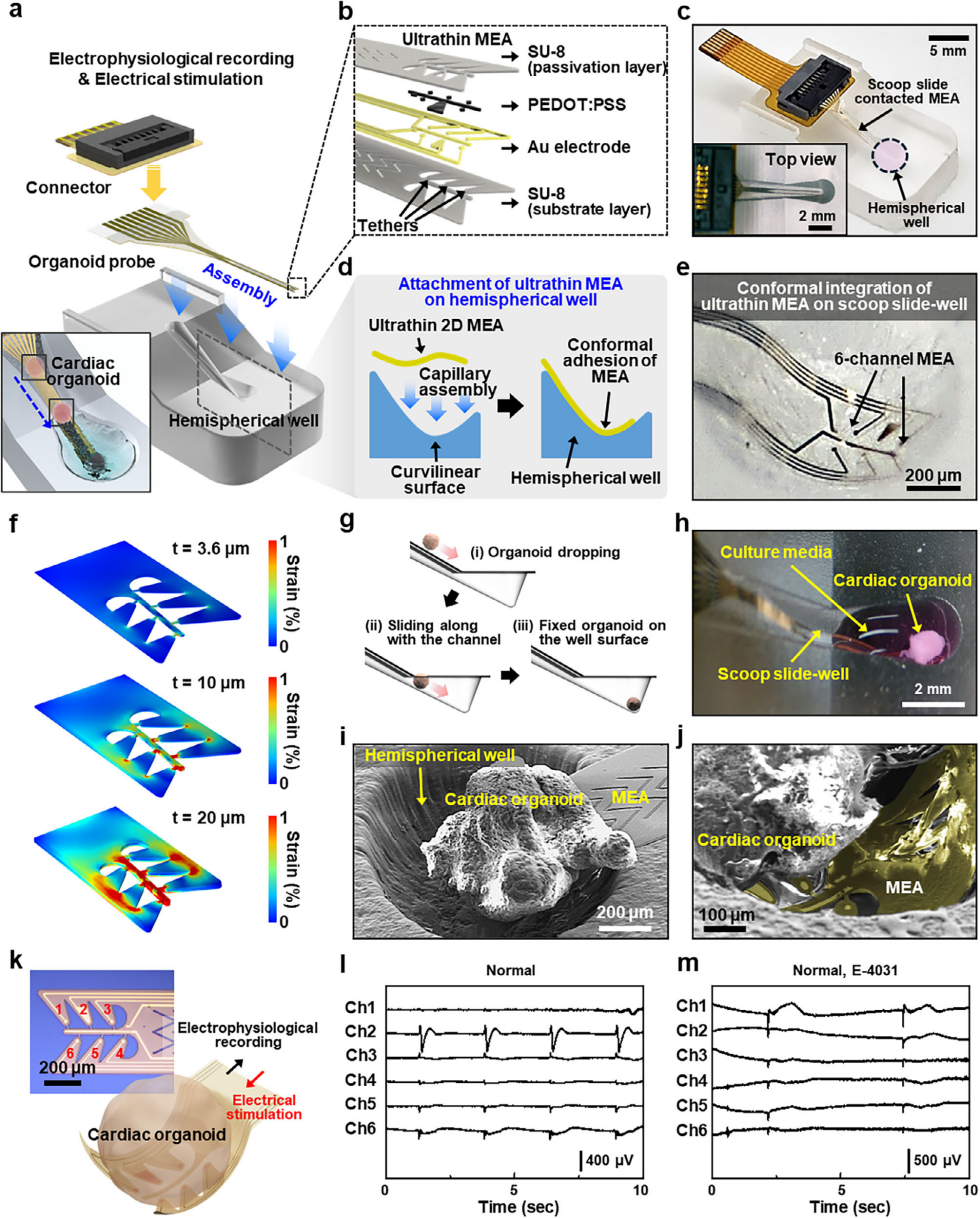
Design, integration, and functional configuration of a deformable MEA platform for 3D cardiac organoid interfacing. (a) Schematic illustration of the ultrathin MEA assembly with a designed scoop slide‐well. (b) Expanded schematic of a multilayered ultrathin MEA, consisting of SU‐8 structural supports serving as substrate and passivation layers that embed Au/PEDOT:PSS electrode array. (c) Photograph of fully assembled platform with MEA conformally aligned to the scoop slide well with a connector for signal input/output (top view inset showing MEA alignment). (d) Capillary‐driven attachment of an ultrathin MEA onto a hemispherical well, enabling conformal adhesion to curved surfaces via van der Waals interaction. (e) Magnified micrograph showing integration of the 6‐channel MEA within the curved region of the hemispherical well, the primary configuration used for systematic studies. (f) FEA results showing a strain distribution along the boundaries of the tether structures as a function of MEA thickness. (g) Schematic of gravity‐guided organoid loading and positioning within the scoop architecture. (h) Magnified micrograph of a cardiac organoid immersed in culture medium and retained at the well center. (i,j) SEM images showing stable, conformal contact between the organoid and MEA on the hemispherical substrate. (k) Schematic illustrating organoid–MEA interface and signal collection across multiple contact points; electrode layout for multi‐site recording and localized stimulation. (l) Representative 6‐channel EFP recordings from a normal CO. m) Representative 6‐channel EFP recordings under drug stimulation with 1000 nm E‐4031.

The platform for electrophysiological signal acquisition in COs (Figure 1a) incorporates a customized scoop slide‐well, an ultrathin MEA fabricated via photolithography, and integrated signal input/output connectors. This architecture enables simultaneous electrophysiological recording and electrical stimulation. Unlike conventional planar MEAs [17], our design employs a simplified assembly process to interface with a 3D hemispherical well, effectively addressing limitations in organoid signal detection. The MEA was fabricated as a multilayered structure optimized for biocompatibility, consisting of SU‐8 photoresist, Au electrodes, and PEDOT:PSS for impedance tuning (Figure 1b; Figure S1) [18]. A key innovation lies in the fabrication process and the culture well design, which together allow stable positioning of the organoids. SU‐8 enables custom micropatterning to achieve precise control over electrode thickness and geometry. Additionally, an insulating SU‐8 layer defines the exposed microelectrode regions, creating a monolithic architecture. Following fabrication on a Si/SiO_2_ wafer (Figure S2a), the MEA was detached from the substrate through selective etching of the SiO_2_ layer to yield a freestanding device. This freestanding MEA was then bonded to a scoop side‐well (i.e., molded from a 3D‐printed template using polydimethylsiloxane, PDMS) to complete the integrated platform (Figure S2b). Figure 1c presents a digital image of the system, with the MEA conformally integrated into the scoop slide‐well. The inset top‐view image highlights the microfluidic channel layout, where precise MEA alignment guides organoids toward the target region via a slide‐like structure. This configuration supports natural settling and consistent contact between the organoids and the electrode array positioned along the curved base of the well.

As illustrated in Figure 1d, a droplet of DI water was introduced at the interface between the MEA and the PDMS well to initiate capillary‐driven adhesion. Upon evaporation, the water droplet generated a robust interfacial force, enabling the MEA to conform to the curved well surface [19]. A critical factor facilitating this conformal adhesion was the design of tether‐like structures in the central region of the MEA. These tethers were engineered to expand laterally upon contact with the well surface, thereby increasing surface compliance and ensuring intimate mechanical coupling. Figure 1e shows a digital image of the MEA integrated into the hemispherical well, conforming to the underlying surface topography (as indicated by the dotted region in Figure 1c). The MEA's ultrathin profile (≈4 µm), combined with its deformable tether structures, facilitated its integration into the electrophysiological measurement system for COs. Based on this demonstration, the specific mechanical mechanism by which these tethers deform (i.e., strain‐induced geometric folding) can be clarified when supported by quantitative modeling. Thus, to quantitatively evaluate the mechanical performance of the ultrathin MEA on curved surfaces, finite element analysis (FEA) was conducted with a specific focus on the central tether regions. The MEA was modeled as a layered structure incorporating the mechanical properties of SU‐8 with material parameters assigned based on literature‐reported Young's moduli and Poisson's ratios. The 460 µm‐wide MEA was virtually conformed onto a hemispherical surface with a diameter of 500 µm, simulating its integration into the hemispherical region of the scoop slide‐well. The simulation domain included the tether structures patterned at the center of the MEA layout. To understand the influence of geometric variations on strain distribution, multiple tether configurations were tested by varying thickness. As presented in Figure 1f, the simulation results revealed a localized concentration of strain along the boundaries of the tether structures, especially in the transition regions between the flexible interconnects and the central contact zone. Notably, tethers with extended arc lengths and reduced thickness exhibited enhanced compliance, effectively distributing mechanical strain and reducing the risk of delamination under curvature‐induced stress. By maintaining a sub‐5 µm thickness, the MEA adhered effectively to the curved surface, even in prototype platforms designed to trap organoids as they roll from the inclined scoop slide (Figure S3). These results validate the mechanical role of the tether structures in supporting conformal adhesion to the hemispheric well by minimizing localized stress and enhancing surface adaptation. The raw signals obtained from the flexible MEA are heavily obscured by environmental noise, primarily power line interference, making it difficult to discern EFPs (Figure S4a). However, after applying our preprocessing pipeline (notch filter combined with DWT denoising), the periodic EFPs are clearly revealed across multiple channels (Figure S4b). This comparison visually demonstrates the efficacy of our denoising steps in recovering high‐fidelity cardiac signals. As depicted in Figure 1g, the inclined plane of the scoop structure facilitated gravitational movement of the organoids toward the central electrode zone, where they settled at the lowest point, immersed in culture medium, optimizing electrical contact and signal acquisition. During the design of the scoop geometry, we considered the typical millimeter‐scale diameter of cardiac organoids and the lateral dimensions of the MEA region to ensure that organoids contacting the inclined surface are funneled into the central trapping zone above the electrodes. This design provides robust self‐aligned positioning that tolerates variations in the initial loading position and slide angle. The scoop slide‐well thereby serves a dual function, enabling both electrophysiological signal recording and electrical stimulation, while also retaining culture medium to sustain organoid viability, as shown in Figure 1h. This careful design allowed for real‐time media exchange or drug delivery during signal acquisition with dynamic monitoring of electrophysiological responses. To avoid organoid displacement caused by vortex effects during fluid exchange, the angled geometry of the slide was utilized for gentle, gravity‐assisted media loading. Figure 1i,j display SEM images of organoid‐MEA contact, revealing robust surface adhesion despite morphological irregularities in the organoid structure.

Figure 1k illustrates the MEA configuration, consisting of six‐channel electrodes arranged to enable electrophysiological recording. Each electrode was 20 µm in diameter and spaced 125 µm center‐to‐center, a design optimized to minimize electrical crosstalk between adjacent sites. Notably, the stimulation electrode was deliberately enlarged to provide broader coverage of the CO surface, thereby ensuring consistent and robust electrical coupling. The lower schematic in Figure 1k also depicts the virtual contact between the CO and the MEA, featuring the distribution of electrode‐organ interface zones. Extracellular field potentials (EFPs) were recorded through the six MEA channels and denoised using discrete wavelet transform (DWT) filtering, as described in the Experimental Section. As shown in Figure 1l, electrophysiological signals from normal COs (i.e., healthy‐donor‐derived COs) were successfully captured across all six channels. The recorded waveforms (each with an amplitude of ≈1 mV) exhibited temporally heterogeneous patterns, indicative of region‐specific differences in ion channel expression within the organoid [20]. To assess the pharmacological sensitivity of the system, we examined the response to E‐4031, a specific hERG channel blocker. Figure 1l,m display representative traces acquired under normal conditions and following 1000 nm E‐4031 treatment. Remarkably, the platform faithfully captured the drug‐induced electrophysiological alterations. While the control group showed characteristic EFP waveforms, the treated group exhibited a prolongation of field potential duration (FPD), reflecting delayed repolarization, which demonstrated the robustness of the MEA design for cardiotoxicity screening.

### 2.2 Characterization of hiPSC‐Derived CO and DMD Genetic Phenotype

The clinical data of the two DMD patients selected for this study are summarized in Table S1. Both patients carried known pathogenic mutations in the *DMD* gene: c.8994C>T (p.R2982^*^) and a frameshift mutation c.6655_6656delGA (p.D2219Ffs^*^3). Electrocardiogram (ECG) recordings revealed distinct clinical phenotypes between two DMD patients: DMD patient 1 exhibited no specific ECG abnormalities, whereas DMD patient 2 presented with a documented history of ventricular hypertrophy (Figure S5). To ensure the genetic background integrity of our models, we performed whole‐exome sequencing (WES) on two normal donors and two DMD patients (Table S2 and Table S3). While benign missense variants in the *DMD* gene were detected in the normal group, high‐impact variants were exclusively identified in the DMD patients, consistent with their clinical diagnoses. Crucially, WES analysis confirmed that no other significant pathogenic mutations associated with cardiomyopathy in the normal group were present, validating these lines as suitable normal and DMD models. Based on this diagnosis, urine‐derived eptithelial cells were isolated from both the two DMD patients and two healthy donors to establish hiPSCs (Figure S6a). Pluripotency was confirmed by the expression of the stem cell markers SOX2 and NANOG (Figure S6b). Furthermore, karyotyping confirmed that the hiPSCs maintained normal chromosomal structures without the abnormalities often associated with long‐term iPSC culture (Figure S6c). Having validated the pluripotency and genomic integrity of these lines, we proceeded to characterize the formation of COs to model in vitro cardiogenesis.

**FIGURE 2:**
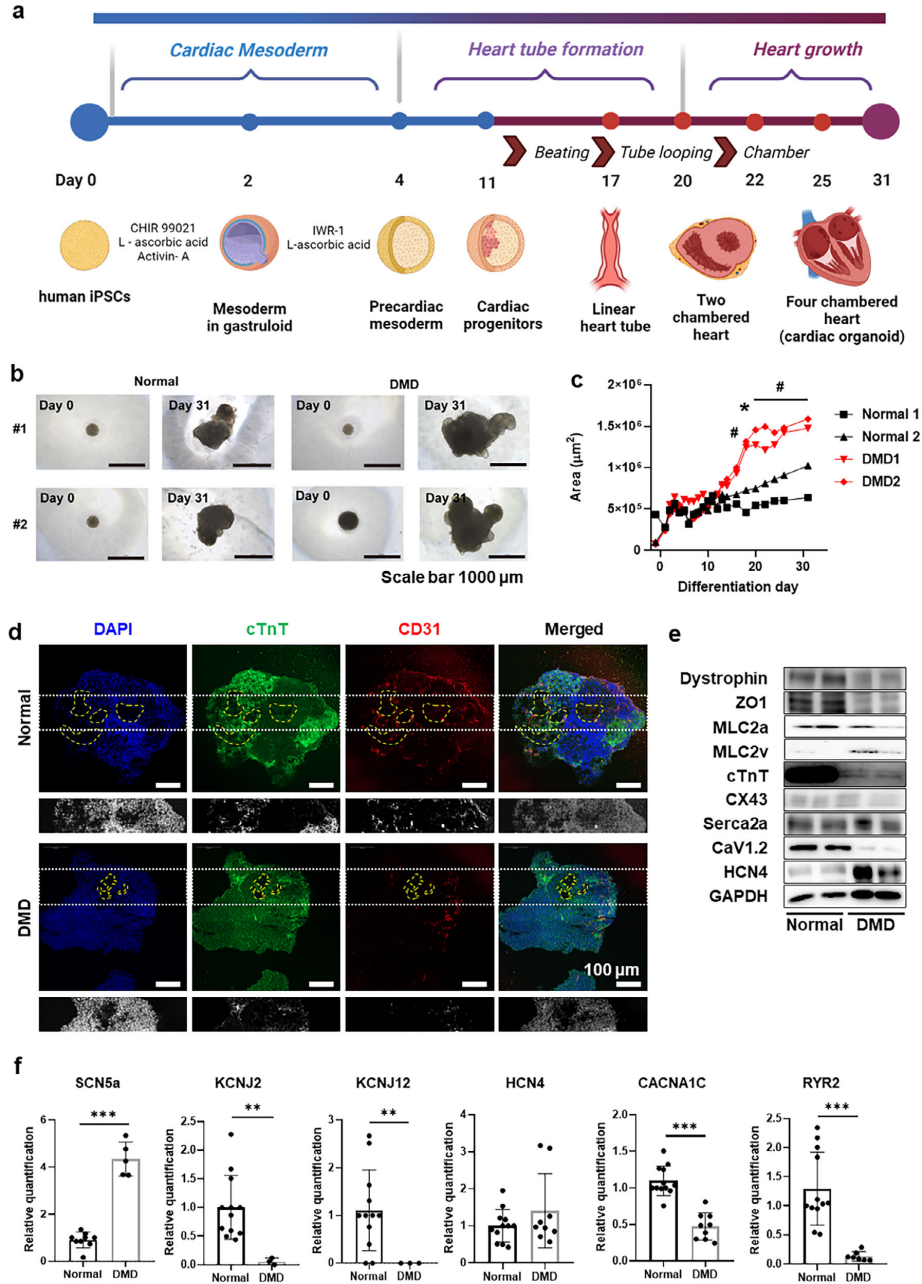
Developmental trajectory and structural characterization of normal and DMD patient‐derived COs. (a) Schematic timeline of CO development from hiPSC‐derived mesoderm to mature heart‐like structures, highlighting distinct phases: cardiac mesoderm induction, heart tube formation, and heart growth. (b) Representative bright‐field images of normal (left) and DMD (right) COs at Day 0 and Day 31. DMD COs display aberrant morphology characterized by enlarged diameter and irregular structure compared to normal COs. (c) Time‐course tracking of COs area, revealing a significant increase in DMD COs compared to normal counterparts at the end point (Day 31); *n* = 20 for each normal group; *n* = 20 for each DMD group. Statistical significance was determined using the non‐parametric Mann–Whitney test (^*^ *p* < 0.01; ^#^ *p* < 0.001). (d) Immunofluorescence images showing co‐staining for nuclei (DAPI, blue), CMs (cTnT, green), and vascular endothelial cells (CD31, red) in Day 25 COs. DMD COs exhibit disorganized CM networks and reduced endothelial signal intensity. (e) Western blot analysis of cardiac‐specific proteins confirming altered expression of structural, functional, and lineage markers in DMD versus normal COs, indicative of impaired cardiac maturation and morphogenesis. *n* = 4 biologically independent pools per group. (f) Quantitative PCR analysis of major cardiac ion channel genes in normal and DMD COs. Relative mRNA expression levels of *SCN5A*, *KCNJ2*, *KCNJ12*, *HCN4*, *CACNA1C*, and *RYR2* were normalized to a housekeeping gene. Notably, *KCNJ2* and *KCNJ12 t*ranscripts were undetectable in DMD COs. Data are presented as mean ± SD (*n* = 12 for normal, *n* = 9 for DMD). Statistical significance was determined using the non‐parametric Mann‐Whitney test (^**^ *p* < 0.01, ^***^ *p* < 0.001).

COs were generated from hiPSCs following the protocol illustrated in Figure 2a. Although differentiation was initiated with an identical starting cell number of 2000 iPSCs per embryoid body (EB) for both groups, a significant morphological disparity emerged by day 31 (Figure 2b; Figure S7). Specifically, DMD COs exhibit a markedly larger size compared to the normal COs from day 12 of differentiation onward (Figure 2c; Figure S7). COs display complex morphological changes during differentiation, progressing through stages of cardiac tube formation, looping, and chamber formation. Throughout this process, the organoids mimic the actual cardiac developmental pathway and develop into a progressively heterogeneous structure (Figure S8). Given that angiogenesis is critical for organoid maturation [21], we evaluated the angiogenic capacity and dystrophin expression profiles of the COs (Figure 2d; Figure S9). Immunostaining for cardiac troponin T (cTnT), a structural marker of CMs, and CD31, a marker of vascular endothelial cells, revealed striking differences between the groups. After 25 days of culture, DMD COs exhibited markedly reduced or absent CD31‐positive vascular structures, indicating impaired angiogenesis. This incomplete structural organization, coupled with the aberrant increase in size, suggests that the DMD COs form disorganized cellular aggregates rather than compact, functional cardiac tissues, reflecting deficient dystrophin expression.

To assess cardiac tissue maturation at the molecular level, we quantified the expression of key markers associated with the cardiac structure and ion channel regulation (Figure 2e; Figure S10). Western blot analysis confirmed the molecular hallmarks of DMD cardiomyopathy in our CO model. Dystrophin deficiency was accompanied by the downregulation of structural markers such as cTnT and ZO‐1, indicating compromised sarcomeric organization and cell‐to‐cell adhesion. Notably, the markedly lower expression of MLC2v in both COs suggests a failure in ventricular‐specific maturation. Furthermore, the altered ion channel profile, characterized by decreased CaV1.2, Serca2a, and elevated HCN4, reflects an electrophysiological immaturity and a potential predisposition to arrhythmic phenotypes, despite the preserved expression of Cx43. Meanwhile, HCN4 expression increased significantly in DMD COs at day 21 but decreased significantly by day 31 (Figure S11). Regarding electrophysiological markers, quantitative PCR (qPCR) revealed distinct remodeling in DMD COs (Figure 2f). Specifically, *SCN5A* mRNA levels were significantly upregulated compared to normal COs. In contrast, genes encoding potassium channels (*KCNJ2, KCNJ12*) and calcium handling proteins (*CACNA1C, RYR2*) were markedly downregulated. Although the expression of the pacemaker gene HCN4 showed no significant difference between the two groups at day 25 (Figure 2f). The dysregulation of ion channel genes may underlie the shortened depolarization phase and prolonged FPD observed in the DMD model. These molecular and structural deviations are not only hallmarks of DMD pathology but also critical determinants of electrode–tissue coupling stability and spatial conduction mapping. Therefore, the following electrophysiological interrogation using the scoop‐integrated MEA directly tests whether the engineered interface can robustly resolve pathological heterogeneity in a mechanically dynamic 3D tissue environment.

### 2.3 Single‐Cell Transcriptomic Profiling of Cellular Heterogeneity in DMD Cardiac Organoids

**FIGURE 3:**
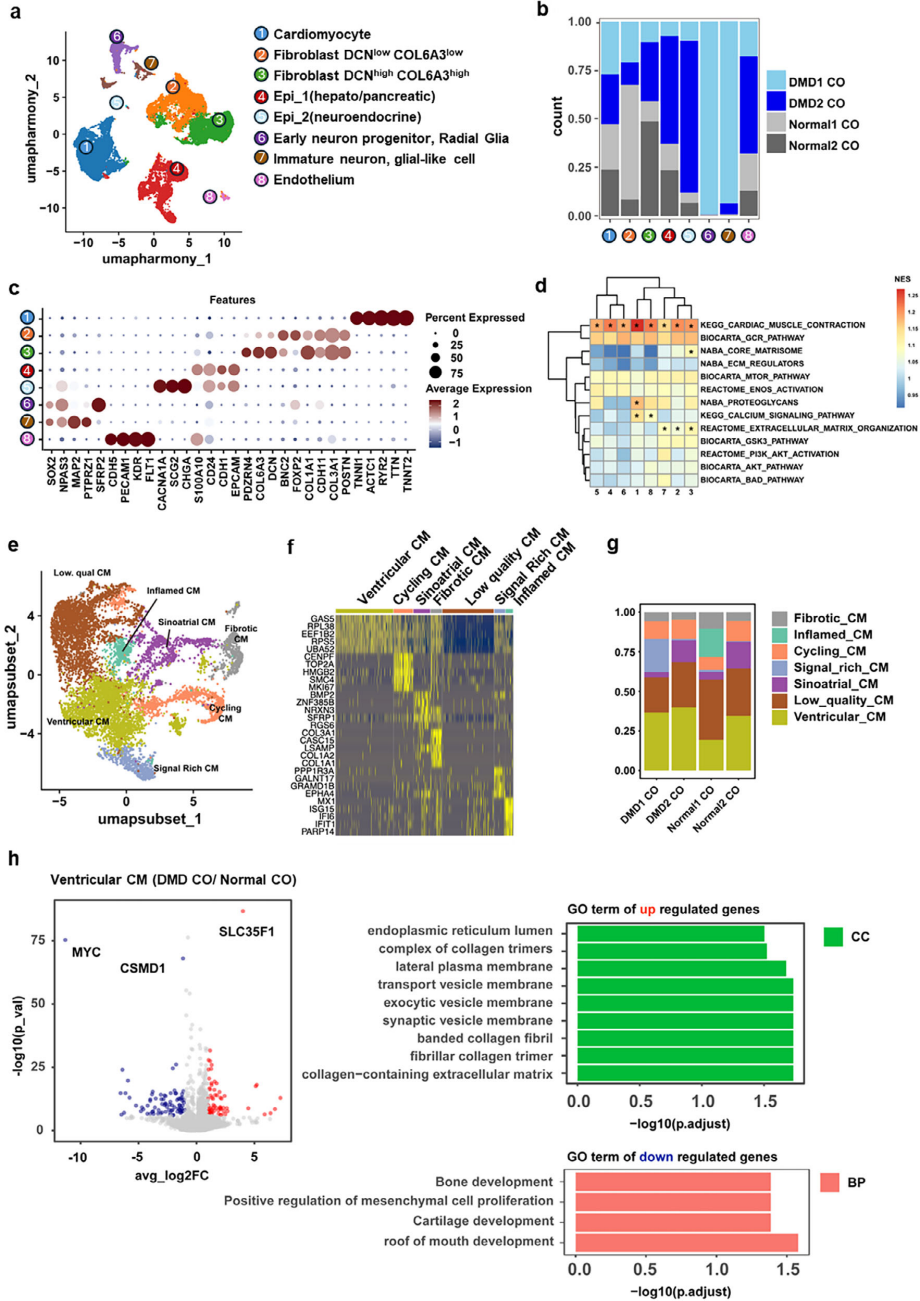
Single‐cell transcriptomic landscape and CM subpopulation analysis in normal and DMD COs. (a) UMAP visualization identifying major cell types, including CM, fibroblast subtypes (DCN^low^ COL6A3^low^, DCN^high^ COL6A3^high^), epi/endothelial lineages, and neuronal progenitors. (b) Stacked bar plot showing the cellular composition of each cluster across normal and DMD samples (*n* = 2 biologically independent pools per group). (c) Dot plot displaying the expression levels of lineage‐specific marker genes used for cluster annotation. (d) Heatmap of Gene Set Enrichment Analysis (GSEA) characterizing the functional identity of each cluster using MSigDB gene sets. Significant enrichment is denoted by asterisks (^*^, *p* < 0.05). (e) UMAP re‐clustering of the CM population, revealing subtypes such as Ventricular, Sinoatrial, Cycling, Fibrotic, and Inflamed CM. (f) Heatmap showing the top differentially expressed markers for each CM subcluster. Marker genes were identified based on a minimum log‐fold change of 0.25, detection in at least 10% of cells within the cluster, and an adjusted *p*‐value ≤ 0.05. The color scale represents the relative expression level (yellow, high expression; dark blue, low expression). (g) Proportional analysis of CM subtypes in normal vs. DMD COs. (h) Differential gene expression analysis of Ventricular CM subset. Left: Volcano plot comparing DMD vs. normal COs, highlighting key differentially expressed genes (e.g., *MYC*, *CSMD1*, *SLC35F1*). Significant DEGs were defined by an absolute log2 fold‐change ≥ 1 and an adjusted *p*‐value ≤ 0.05, calculated using the Benjamini–Hochberg method for multiple testing correction. Right: Gene Ontology (GO) enrichment analysis revealing the upregulation of ECM‐related terms (CC, cell component) and down‐regulation of developmental terms (BP, biological process) in DMD COs. Significantly enriched GO terms were defined by an adjusted *p*‐value ≤ 0.05, calculated using the Benjamini–Hochberg method for multiple testing correction.

To investigate cellular heterogeneity and intercellular signaling, we performed single‐cell RNA sequencing (scRNA‐seq) on normal and DMD COs. Samples were collected from two donors per group, with more than 40 organoids pooled per sample to ensure representation. Uniform Manifold Approximation and Projection (UMAP) visualization identified eight distinct cell populations: CMs, two fibroblast subtypes, two epithelial subtypes, endoderm, neuro‐ectoderm, and endothelial cells (Figure 3a). The cellular distribution across samples is presented in Figure 3b. Regarding the CM proportion (Group 1), while CM subtype was consistently detected across all samples, we observed significant donor‐to‐donor variation rather than a consistent trend distinguishing normal from DMD groups. Cellular identities were characterized by visualizing the expression levels of the top five marker genes for each cluster (Figure 3c). For a more comprehensive analysis, a detailed transcriptomic landscape is provided in Figure S15a, and the precise cell type composition ratios are listed in Table S5. Subsequently, Gene Set Enrichment Analysis (GSEA) was performed to investigate functional differences between the groups (Figure 3d; Figure S15b). The analysis revealed significant variations in pathways associated with cardiac muscle contraction, proteoglycans, calcium signaling, and extracellular matrix (ECM) organization. To further dissect the heterogeneity within the CM population, we performed sub‐clustering analysis, which resolved seven distinct subtypes: ventricular CM, cycling CM, sinoatrial CM, fibrotic CM, low quality CM, signal‐rich CM, and inflamed CM (Figure 3e). The identity of each subcluster was validated through the expression of the top five marker genes (Figure 3f). Although the proportional distribution of these subtypes showed donor‐dependent variation (Figure 3g), we focused our downstream analysis on the ventricular CMs, the major functional unit, to identify disease‐specific transcriptomic alterations.

In addition to ionic remodeling, an altered ECM composition and abnormal cell proliferation are well‐documented features of DMD pathology [22]. Specifically, increased fibrosis and ECM remodeling characterize skeletal muscle degeneration in DMD [23]. Consistent with this, our differential expression analysis between DMD and normal COs within the ventricular CM subset revealed distinct molecular signatures (Figure 3h). Gene Ontology (GO) analysis demonstrated that genes upregulated in DMD ventricular CMs were significantly enriched in terms related to ECM and collagen organization, such as “collagen‐containing extracellular matrix” and “fibrillar collagen trimer.” These findings suggest that DMD ventricular CMs intrinsically acquire a profibrotic transcriptomic profile, potentially contributing to the pathological stiffness and dysfunction of the tissue.

To investigate the genetic basis of the observed cardiac waveform alterations, we analyzed the distribution of genes defining ventricular, atrial, and pacemaker lineages (Figure S18a). The analysis revealed that each CM subtype exhibited distinct gene signatures. Assessment of ventricular (Figure S18b), atrial (Figure S18c), and sinoatrial markers (Figure S18d) allowed for a clear visualization of cellular composition. This transcriptomic classification was further corroborated by immunofluorescence staining. While normal COs displayed a robust and uniform distribution of cardiomyocytes around with nascent tubular structures, DMD COs exhibited fragmented, localized clusters of ventricular and atrial subtypes (Figure S19). To elucidate the molecular basis of the waveform alterations, we examined the expression of gap junction genes (Figure S20). *GJA1* (Cx43) was predominantly expressed in ventricular CMs, whereas *GJA5* (Cx40) showed broad expression without a distinct expression pattern. Notably, *GJC1* (Cx45) was specifically enriched in Sinoatrial CMs. These findings confirm that while each CM subtype maintains its distinct gap junction profile, the structural fragmentation and disorganized spatial distribution observed in DMD COs may disrupt these subtype‐specific electrophysiological properties, ultimately leading to the recorded waveform alterations.

Finally, we examined the expression profiles of fundamental ion channels—sodium, calcium, and potassium—across the whole CO population (Figure S21a). While mature CMs were generally enriched for calcium and potassium channels, a specific comparison revealed that DMD COs exhibited significantly reduced calcium channel expression compared to normal COs (Figure S21b). To functionally validate calcium‐handling properties, we performed Fluo‐4 AM imaging (Figure S21c). Normal COs displayed a longer beat period and a prolonged spike rise time. In contrast, DMD COs exhibited a distinct calcium handling profile characterized by a shorter spike rise time and higher spike amplitude, consistent with the upregulation of sodium channels observed in Figure 2f. However, despite the rapid upstroke kinetics driven by sodium channels, the widespread downregulation of calcium and potassium channels resulted in an overall immature and unstable beating pattern. Collectively, the single‐cell transcriptomic and calcium‐handling analyses provide a mechanistic substrate for the spatially heterogeneous and temporally unstable waveforms interrogated by the six‐channel MEA. This integrative evidence functionally validates the necessity of multipoint conformal interfacing under 3D boundary conditions, rather than relying on planar single‐site recordings that are prone to contact‐dependent artifacts.

### 2.4 Investigation of Drug Effects on Electrophysiological Signals in DMD COs

**FIGURE 4:**
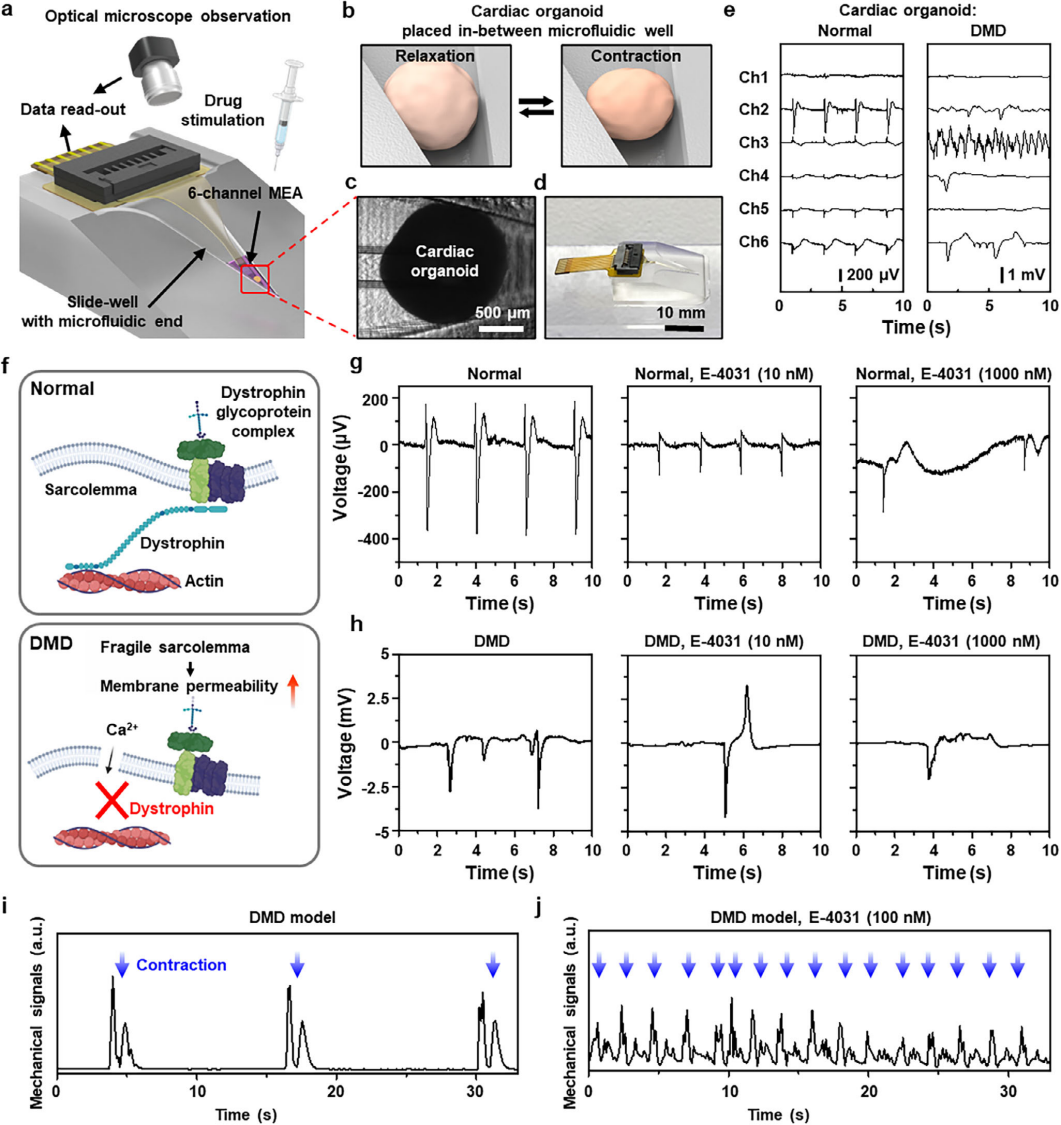
Functional assessment and drug response profiling in cardiac organoids using slide‐well‐based MEA platform. (a,b) Schematic illustration of a cardiac organoid positioned in an alternative tapered microfluidic‐end configuration for EFP recording and real‐time video acquisition, enabling minimal fluid volume positioning and multiple organoid loading. (c,d) Representative micrographs showing cardiac organoid alignment within the curved well. (e) Comparison of representative EFP traces between normal and DMD COs is shown on an identical time scale. (f) Schematics depicting the pathophysiological consequences of dystrophin deficiency. (g,h) Representative EFP traces showing electromechanical coupling with E‐4031dose dependent treatment (g) and decoupling in DMD COs (h). (i,j) Representative traces of DMD COs under E‐4031 challenges, exhibiting severe electromechanical dissociation. The blue arrows indicate the contraction cycle before and after drug stimulation, indicating heightened arrhythmogenic susceptibility and instability in DMD COs.

Using the scoop slide integrated with the MEA platform, we performed simultaneous recordings of electrophysiological signals and real‐time video‐based motion analysis during contraction and relaxation cycles. As illustrated in Figure 4a, the microfluidic‐structured guidance well was specifically engineered to position organoids between its lateral walls, enabling stable EFP measurements via a six‐channel MEA. The experimental configuration is depicted in Figure 4b, illustrating how the millimeter‐scale CO are loaded into the slide‐well featuring a microfluidic inlet. As the organoid is gently introduced, gravity directs it into the narrow capture zone. In the bright‐field image, a single CO is shown stably mounted on the six‐channel MEA between the two PDMS walls, with its diameter closely matching the well width to prevent lateral drift during contraction (Figure 4c). Notably, the versatility of this tapered microfluidic channel design was demonstrated by the capability to load two COs that fuse and beat simultaneously, enabling concurrent motion measurements (Movie S1). Furthermore, the fully assembled module was mounted on a custom holder with a standard connector interface (Figure 4d), a design that facilitates the rapid swapping of COs without disturbing electrical contacts. This combination of passive hydrodynamic trapping and precise geometric confinement ensured secure, high‐fidelity recordings of both EFPs and mechanical motion, minimizing artifacts even during drug‐induced arrhythmic events. Applying this system to the DMD CO model, we successfully captured multichannel EFP waveforms from the DMD COs, which are presented alongside representative traces from normal COs (Figure 4e). This electromechanical uncoupling suggests a disruption in signal propagation, likely stemming from dystrophin deficiency and the associated sarcolemma instability. At the tissue level, while gap junction‐mediated coupling is essential for synchronized propagation, the excessive intracellular Ca^2^^+^ accumulation caused by membrane instability may impair gap junction function or downregulate connexin expression, thereby disrupting conduction pathways and leading to the aberrant EFP signatures observed in DMD COs. The recorded signals exhibited heterogeneous profiles across the six channels, characterized by varying amplitudes and waveform morphologies. These variations reflect the spatial complexity of electrophysiological activities within the 3D COs, confirming the platform's capability to resolve spatially distinct features essential for analyzing complex arrhythmic events in pathological models such as DMD.

As schematically illustrated in Figure 4f, DMD arises from pathogenic mutations, deficiency, duplication, or point mutations in the *dystrophin* gene. In healthy myocardium, dystrophin, along with the dystrophin‐glycoprotein complex (DGC) stabilizes the sarcolemma during contraction, thereby maintaining intracellular calcium (Ca^2^^+^) homeostasis concentrations by stabilizing the cell membrane, thereby enabling CMs to withstand continuous mechanical stress. In contrast, dystrophin deficiency in DMD patients renders the myocardial cell membrane fragile. Consequently, the membrane becomes susceptible to micro‐ruptures under repeated mechanical stress, a phenomenon known as “fragile sarcolemma.” This mechanical instability exacerbates pathological Ca^2^^+^ influx, triggering dysregulated calcium signaling cascades. The ensuing calcium overload damages critical organelles such as mitochondria and activates cell death pathways, including autophagy and apoptosis, ultimately causing myocardial fiber degeneration and cardiomyopathy [24]. We first examined the dose‐dependent response of normal COs to E‐4031 (Figure 4g). At baseline, normal COs exhibited regular pacemaker firing and subsequent muscle depolarization. Treatment with a low dose of E‐4031 (10 nm) resulted in a distinct prolongation of the repolarization phase without disrupting the regular rhythm, a characteristic sign of hERG channel inhibition. At a high concentration (1000 nm), the normal COs displayed severe field potential instabilities, characterized by early afterdepolarization‐like triggered activities and irregular beating rhythms; these extracellular waveform alterations are consistent with a profound blockade of the hERG‐mediated potassium current. In contrast, DMD COs exhibited a markedly heightened sensitivity to E‐4031 (Figure 4h). Even at the low concentration (10 nm), where normal COs maintained a regular rhythm, DMD COs developed severe arrhythmic events, characterized by prominent afterdepolarization‐like deflections in extracellular recordings and irregular inter‐spike intervals. This suggests that the baseline electrophysiological stability of DMD COs is already compromised, making them prone to pro‐arrhythmic events even under mild pharmacological stress. Furthermore, exposure to the high dose (1000 nm) in DMD COs led to a drastic deterioration of signal integrity, exhibiting chaotic electrical activity distinct from the patterns observed in normal controls. These findings demonstrate that the DMD COs on our platform faithfully recapitulate the pathological phenotype of electrical instability and drug hypersensitivity, validating the system's utility for disease modeling and safety pharmacology screening. Finally, a key advantage of this platform is its compatibility with optical imaging, which allows for precise analysis of contraction and relaxation dynamics using real‐time video observation. We quantified the mechanical motion of the DMD COs by analyzing pixel intensity variations within a fixed region of interest (ROI). The detailed process of analyzing mechanical signals using experimentally observed video data is described in the Experimental section. As shown in Figure 4i, prior to drug stimulation, the DMD COs exhibited distinct motion profiles representing baseline mechanical beats. However, following exposure to E‐4031 (100 nm), the contraction pattern was drastically altered (Figure 4j; Movie S2). The mechanical analysis revealed a significant acceleration in the contraction cycle, indicating a drug‐induced arrhythmic response. Specifically, the motion traces displayed characteristic double‐peak waveforms, where the primary contraction peak is followed by a slightly lower relaxation peak. Furthermore, we observed frequent fine irregular fluctuations between these major peaks. These mechanical instabilities likely reflect the chaotic physiological state induced by the hERG blockade in the dystrophic tissue. This series of experimental approaches demonstrates the utility of our MEA‐integrated biointerface platform for detecting drug‐induced arrhythmogenic responses not only electrophysiologically but also mechanically. The system's ability to capture these complex dynamics highlights its potential for comprehensive cardiotoxicity screening and disease modeling in DMD‐related cardiac dysfunction.

Crucially, CO models enable researchers to dissect the biological mechanisms underlying cardiac contraction, revealing significant differences compared to hiPSC‐derived CMs cultured on traditional 2D MEA [25]. A representative application is the use of E‐4031, a selective human Ether‐à‐go‐go‐Related Gene (hERG) channel blocker commonly used in the International Conference on Harmonization (ICH)‐recommended in vitro assay to assess pro‐arrhythmic risk, specifically the TdP assay [26]. In this study, both normal and DMD COs, as well as their 2D CM counterparts, exhibited distinct electrical and mechanical responses following E‐4031 treatment (Figure S22). Stimulation of normal COs with 10 nm E‐4031 induced significant arrhythmic patterns. These were characterized by a marked increase in pacemaker rate and a decrease in field potential amplitude, resembling TdP‐like ventricular tachycardia.

## 3 Discussion

The primary objective of this study was not to further delineate cardiac organoid development per se, but to establish a mechanically and geometrically adaptive bioelectronic interface capable of resolving electrophysiological phenotypes in structurally heterogeneous 3D tissues. The extensive structural and transcriptomic characterization presented herein serves as a validation framework to confirm that the electrical signatures captured by the scoop‐integrated deformable MEA reflect intrinsic tissue‐level remodeling rather than interface artifacts. As such, organoid heterogeneity constitutes the engineering boundary condition under which the performance of the MEA platform must be evaluated. In this study, we established a robust electrophysiological assessment platform for COs derived from healthy and DMD‐patient iPSCs by integrating a flexible, deformable MEA within a specialized scoop‐slide structure. A critical challenge in organoid‐based modeling has been the geometric mismatch between the grid, planar electrodes, and the irregular, 3D morphology of COs, which often compromises signal fidelity [27]. Our design specifically addresses this limitation; the scoop‐shaped well guides the organoid into a defined position, while the deformable MEA ensures conformal contact with the tissue surface. This enables direct, label‐free measurement of key parameters such as arrhythmia generation and conduction velocity, going beyond simple surrogate markers like calcium imaging [28].

While the supplementation of exogenous ECM components is considered critical for organoid self‐organization [29, 30, 31], our scaffold‐free system successfully drives an intrinsic developmental trajectory without relying on external supports like Matrigel. The heart tube and looping structures formed during this process closely mimic the structural organization of the in vivo embryonic heart [9]. Crucially, in DMD COs, this ECM‐independent approach unmasks abnormal endogenous ECM accumulation and structural heterogeneity, which directly manifest as conduction heterogeneity [32]. Within this morphological complexity, our platform's six‐channel MEA successfully captures spatially distinct electrical fields within this morphological complexity, enabling the precise differentiation of pathological signals within the disease model.

Using the selective hERG blocker E‐4031 as a repolarization stressor, we observed a clear divergence in drug responses between control and DMD organoids. Control COs exhibited the expected dose‐dependent prolongation of EFP‐derived repolarization indices (e.g., FPD/FPDc when measurable) with preserved rhythmicity. In contrast, DMD COs frequently transitioned to early electrical instability and arrhythmia at low concentrations, which could preclude reliable interval‐based measurements once distinct repolarization features were lost. Consistent with this functional hypersensitivity, our scRNA‐seq analysis revealed comparable KCNH2 transcript patterns between normal and DMD cardiac subtypes (Figure S23). This suggests that the observed phenotype is not driven by a primary deficiency in hERG expression, but rather by increased repolarization vulnerability, which is likely exacerbated by the concurrent downregulation of inward rectifier potassium channels (e.g., KCNJ2) as observed in our transcriptomic profiling. Accordingly, we report repolarization‐related metrics only where waveform morphology supports interval quantification, treating rhythm instability itself (arrhythmia incidence/burden and time‐to‐instability) as a complementary quantitative phenotype under stress conditions. While E‐4031‐induced instability indicates reduced repolarization reserve, direct attribution to KCNH2/I_Kr_ dysfunction remains beyond the resolution of our current dataset.

To acquire these high‐fidelity signals, our platform employs a specialized biointerface that achieves conformal adhesion of individual organoids to electrodes through controlled capillary forces and culture‐medium volume modulation [33]. While this strategy yields higher signal‐to‐noise ratios and improved spatial resolution compared to the stochastic, bulk‐culture configurations of conventional 2D MEAs, it introduces a bottleneck in data scalability. The current manual loading and alignment workflow, although accurate, is labor‐intensive and can introduce inter‐operator variability, which limits rapid collection of the large datasets—often on the order of thousands of independent recordings—typically required to develop and validate robust machine‐learning or artificial‐intelligence pipelines [34]. Beyond throughput, the pathological heterogeneity of signals derived from DMD COs poses an additional computational challenge [35]. In normal COs, automated peak‐detection and feature‐extraction algorithms generally assume quasi‐stationary and periodic cardiac cycles. In contrast, the fragmented and temporally non‐stationary waveforms frequently observed in DMD COs can violate these assumptions and reduce the reliability of standard EFP feature extraction [32]. The absence of stable electrophysiological baselines in diseased models may therefore require expert‐guided annotation to minimize error propagation during automated analysis [34]. Accordingly, while our platform enables deep phenotyping of individual 3D tissues, advancing toward AI‐enabled, high‐throughput screening will benefit from integrating automated microfluidic positioning and developing bespoke, noise‐tolerant algorithms capable of handling non‐linear dynamics in diseased COs.

## 4 Conclusion

In summary, this study establishes a novel bioelectronic interface that effectively bridges the geometric and functional gap between rigid electronics and soft 3D tissues. By integrating a deformable MEA within a specialized scoop‐slide architecture, we achieved high‐fidelity, real‐time electrophysiological profiling of patient‐derived cardiac organoids, overcoming the spatial limitations inherent to conventional 2D systems. Utilizing this platform, we successfully dissected the pathophysiology of DMD, revealing that dystrophin deficiency leads to profound electrophysiological instabilities and structural disorganization. Crucially, our system demonstrated the capability for targeted high‐throughput drug screening, validating its utility in capturing complex disease phenotypes such as drug‐induced arrhythmias within a physiologically relevant 3D microenvironment. While our platform significantly enhances signal acquisition, challenges related to the inherent heterogeneity of 3D organoids remain. The current fixed‐electrode configuration, although optimized for standard organoid sizes, may require further refinement to accommodate extreme morphological variations, which could affect signal resolution in localized regions. Addressing these variables represents a key avenue for future optimization. Looking forward, the next generation of this platform will aim to incorporate adaptive, stretchable sensor arrays to ensure dynamic conformity with growing tissues. Furthermore, integrating multimodal sensing capabilities, correlating extracellular field potentials with intracellular calcium dynamics or contractile force, will provide a holistic view of electromechanical coupling. Ultimately, this evolved biointerface holds the potential to become a cornerstone technology for cardiac precision medicine, enabling more accurate disease modeling and the acceleration of therapeutic discovery.

## 5 Experimental Section

### 5.1 Materials

StemFlex Medium (A3349401), Accutase (A1110501), Essential 8 (A1517001), Trypsin‐EDTA 0.25% (25200‐056), Trypsin‐EDTA 0.05% (25300‐054), TrypLE TM (12605‐010), fetal bovine serum (16000‐044), RPMI1640 no glucose (11879‐020), DMEM/F12 (11330‐032), mitoSOX (Red mitochondrial superoxide indicator; M36008), and Cytotune‐iPSC2.0 Senai Reprogramming Kit (A16517) were purchased from Thermo Fisher Scientific (Waltham, MA). D‐PBS (LB001‐01) and RPMI1640 (LM011‐01) were purchased from Welgene (Daegu, Republic of Korea). Corning Matrigel hESC‐Qualified Matrix LDEV‐Free (354277), Matrigel matrix GFR (354230) were purchased from Corning Life Sciences (Tewksbury, MA). Deoxyribonuclease 1 (D4263), Pluronic F‐127 (P2443), Albumin human (A9731), L‐ascorbic acid 2‐phosphate (A8960), and Y27632 (688000) were purchased from Sigma–Aldrich (St. Louis, MO). CHIR99021(S2924), IWR‐1‐endo (72562) were purchased from Selleck Chemicals (Houston, TX, USA). L‐Lactic acid (129‐02666) was purchased from FUJIFILM Wako chemical Industries, Ltd (Osaka, JAPAN). Recombinant Activin A (120‐14E) was purchased from Peprotech (Cranbury, NJ, USA). 96 well plate Round‐bottom (34296) was purchased from SPL Life Sciences (Pocheon, Republic of Korea).

### 5.2 MEA Fabrication

Flexible MEA were produced using standard photolithographic techniques on a Si wafer substrate with a thermal SiO_2_ overlayer (i.e., Si/SiO_2_). Photomask patterns were generated in CAD and transferred to a bilayer of photoresist via mask‐alignment. Initially, an ≈2 µm SU‑8 2002 layer (MicroChem, MA, USA) was spin‑coated onto the Si/SiO_2_ substrate (2000 rpm, 60 s) to serve as a sacrificial etch layer. This photoresist film was exposed to UV light (≈60 mJ cm^−^^2^) through a chromium photomask, developed to eliminate unexposed regions, and then hard‑baked at 180°C for 30 min. Subsequently, a metal film (10 nm Cr/100 nm Au) was thermally evaporated and patterned by applying a GXR 601 positive resist, followed by selective etching of Au and Cr to define the microelectrode traces. Insulating structures were formed by spin‑coating and patterning a second SU‑8 layer identical to the first. Finally, the freestanding MEA were released from the mother substrate by immersing in buffered oxide etchant (BOE) to dissolve the SiO_2_ layer, then rinsed and soaked in deionized water for 24 h to remove residual etchant (Figure S2). All organoid‐facing surfaces are composed of SU‐8, Au, PEDOT:PSS, and PDMS, which are widely used as biocompatible materials in neural implants and organ‐on‐chip platforms, supporting the stability and safety of the device architecture for acute recording applications. As this platform is primarily designed for acute extracellular field potential (EFP) recordings rather than chronic electrical stimulation, we prioritized optimizing impedance reduction via PEDOT:PSS coating over maximizing long‐term charge storage capacity. The platform was implemented with two slide‐well geometries sharing the same MEA architecture: a hemispherical scoop design used for the systematic studies, and a tapered microfluidic‐end employed for the specific experimental setup shown in Figure 4.

### 5.3 Clinical Information

The patient is clinically diagnosed with Duchenne muscular dystrophy muscle biopsy with dystrophin stain, MLPA analysis of the DMD gene resulted in negative. High serum creatinine kinase, weakness, calf muscle pseudohypertrophy, positive Gowers sign. The *DMD* gene was analyzed by PCR and next‐generation sequencing of both DNA strands of the entire coding region and the highly conserved exon‐intron splice junctions. PCR‐based amplicon library production was utilized. We have a minimum coverage of 30x for every amplicon. The reference sequence is DMD: NM_0045006.2. Table S1 shows a detailed description of the detected variant.

### 5.4 Generation of hiPSC from Urine Samples

We obtained urine from a normal individual and a DMD patient to isolate epithelial stem cells [21]. Duchenne Muscular Dystrophy human iPSC was generated from DMD patient urine's epithelial cells appearing a nonsense mutation in exon 60 (c.8994 C>T (p.R2982^*^) of the DMD gene that leads to a premature stop codon (IRB Approval of Pusan National University Yangsan Hospital_#L_2021_34). The healthy donor urine (normal) was obtained from IRB approval in Pusan National University (PNUIRB/2021_25_BR). We generated iPSC lines by SeV‐based reprogramming kits (Invitrogen; CytoTune‐iPS 2.0 Sendai Reprogramming kit). Human iPSC lines were cultured feeder‐free on Matrigel hESC‐qualified matrix LDEV‐Free in Essential 8 medium on early stage (by passage 10) and Stem flex medium at 37°C and 5% CO_2_ incubator. Colonies were passaged by 0.5 mm EDTA in PBS‐based solution.

### 5.5 Finite Element Analysis of MEA

The strain distribution of the MEA was calculated using the COMSOL Multiphysics software (COMSOL, Inc., Burlington, MA, USA). To simulate the strain distribution when the MEA is conformally attached to the hemispherical well, the displacement was adjusted so that the tether structure region can be attached to the hemispherical surface with a diameter of 500 µm. The tether region of the MEA was subjected to a vertical displacement of approximately 100 µm. The detailed modeling dimensions and displacement information are described in Figure S26.

### 5.6 Analyses of EFPs and Contraction/Relaxation of the CO

These analyses were performed in MATLAB (R2020b, MathWorks, Natick, USA). To remove power line noise between 59 and 61 Hz, a fourth‐order Butterworth notch filter was utilized. The EFPs were downsampled to 500 Hz for analysis and filtering. EFPs were denoised using the discrete wavelet transform with coif5 wavelets and soft thresholding based on the rigrsure method. To analyze the contraction/relaxation dynamics of the CO, real‐time video‐based monitoring was performed by acquiring grayscale images at 0.15 s interval. Region of interest (ROI) was manually defined using the *drawrectangle* function, and inter‐frame pixel differences within the ROI were computed using the *imabsdiff* function. The resulting time‐series signals reflected mechanical activity, where each pair of peaks corresponded to a contraction and relaxation of the CO.

### 5.7 Single‐Cell RNA Sequencing and Data Pre‐Processing

Single‐cell RNA sequencing (scRNA‐seq) libraries were prepared using the Chromium GEM‐X Single Cell 3’ Reagent Kits v4 (10X Genomics), following the manufacturer's instructions. Cells from two DMD patients (DMD1, DMD2) and two normal controls (Normal1, Normal2) were pooled prior to loading, with a targeted cell recovery of 20 000 cells. Raw sequencing data were aligned and quantified using CellRanger (10X Genomics) [36]. To correct for ambient RNA contamination and background noise, CellBender [37] was applied to the raw feature‐barcode matrices. To facilitate sample demultiplexing, Whole Exome Sequencing was performed to establish donor‐specific genotypes by leveraging the SureSelect XT HS2 DNA Reagent Kit (Agilent Technologies). Variant calling followed the GATK (v 4.1.6.0) Best Practices workflow [38]. Raw reads were aligned to the GRCh38 reference genome using BWA‐MEM [39]. Post‐alignment processing included duplicate marking and Base Quality Score Recalibration (BQSR). Subsequently, variant calling was performed using HaplotypeCaller in GVCF mode, followed by multi‐sample joint genotyping via GenomicsDBImport and GenotypeGVCFs. High‐quality SNPs were retained based on the following filtering criteria: allele frequency between 0.05 and 0.95, Genotype Quality (GQ) greater than 20, Quality by Depth (QD) greater than 10, and an Alternate Allele Fraction greater than 0.2. These filtered SNPs were subsequently utilized in Demuxlet [40] to assign cell barcodes from the scRNA‐seq data to their respective donors and to identify doublets.

### 5.8 Quality Control and Cell Type Annotation

Downstream analysis was performed using Seurat (v5) in R [41]. The CellBender‐corrected gene expression matrices were initialized as Seurat objects. Incorporating the Demuxlet results, we retained only cells classified as singlets that matched the specific donor identities. Quality control filters were applied to remove low‐quality cells and potential multiplets: cells with nFeatrue_RNA <400, nCount_RNA >15 000, and percent.mt > 10% were excluded. Following filtration, datasets from normal and DMD samples were merged. Gene expression counts were normalized using the LogNormalize method with a scale factor of 10,000, and highly variable features were identified. To correct for batch effects and integrate the datasets, we employed Harmony [42] using the top 30 principal components. Cluster‐specific marker genes were identified using the FindAllMarkers in Seurat, selecting positively expressed markers with a minimum log‐fold change threshold of 0.25 and detection in at least 10% of the cells within the target cluster.

### 5.9 Functional Enrichment and Differential Expression Analysis

To elucidate the biological functions associated with each cell type, Gene Set Enrichment Analysis (GSEA) was performed. Reference gene sets were retrieved for Homo sapiens using the “msigdbr” package [43]. Genes were ranked based on their average expression levels within each cluster, and enrichment scores were calculated using the “fgsea” package [44] with 10 000 permutations.

Differentially expressed genes (DEGs) between DMD and normal samples within specific clusters were identified using the FindMarkers function. Significant DEGs were defined as genes with an absolute log2 fold‐change (|log_2_FC| ≥ 1) and an adjusted p‐value ≤ 0.05. The differential expression results were visualized using a volcano plot. Subsequently, to interpret the biological significance of these DEGs, Gene Ontology enrichment analysis was conducted using the clusterProfiler package [45] with the org.Hs.eg.db database [46].

### 5.10 Live‐cell Calcium Imaging

To evaluate the functional calcium handling properties of normal and DMD COs, live‐cell calcium imaging was performed using the cell‐permeant fluorescent indicator Fluo‐4 AM (Thermo Fisher Scientific). The COs were incubated with 2 mm Fluo‐4 AM in culture medium for 30 min at 37°C in a humidified atmosphere with 5% CO_2_. Following incubation, the organoids were washed twice with DPBS to remove excess extracellular dye. After washing, the COs were maintained in a specialized imaging medium consisting of RPMI 1640 supplemented with B‐27 supplement and 0.05% (w/v) Pluronic F‐127. Time‐lapse calcium imaging was conducted using a Dragonfly 502w (Andor Technology), a multifunctional fast spinning‐disk confocal microscope system. To capture the rapid calcium transient kinetics of the organoids, images were acquired at a temporal resolution of 33 frames per second (fps) for a total duration of 1 min per sample.

### 5.11 Statistical analysis

All statistical analyses were performed using GraphPad Prism (version 8.0.1) and R (versions 4.4.0 and 4.4.1). Quantitative data are presented as mean ± standard deviation (SD) from at least three independent experiments, unless otherwise stated. The exact sample size (*n*) for each analysis is provided in the corresponding figure legend. Statistical significance between groups was evaluated using appropriate tests, including Student's *t*‐test, the Mann–Whitney U test, and one‐way or two‐way analysis of variance (ANOVA) followed by appropriate post hoc multiple‐comparison tests, as specified for each experiment. A two‐sided significance threshold of *p* < 0.05 was applied, and statistical significance is indicated as follows: ^*^ *p* < 0.05, ^**^ *p* < 0.01, and ^***^ *p* < 0.001. Detailed procedures for data preprocessing, outlier exclusion, and the statistical criteria used for single‐cell transcriptomic and bulk RNA‐sequencing analyses are provided in the Supporting Information.

## Conflicts of Interest

The authors declare no conflicts of interest.

## Supporting information




**Supporting File 1**: advs74894‐sup‐0001‐SuppMat.docx.

**Supporting File 2**: advs74894‐sup‐0002‐MovieS1.mp4.

**Supporting File 3**: advs74894‐sup‐0003‐MovieS2.mp4.

## Data Availability

The data that support the findings of this study are available in the supplementary material of this article.
