# Supplementary material for: Conformable Microelectrode Arrays Integrated with a Scoop‐Shaped Slide‐Well for Dynamic Electrophysiological Profiling of Patient‐Derived Cardiac Organoids
Source: Adv Sci (Weinh). 2026 Mar 30;13(31):e14365. doi: 10.1002/advs.202514365 (PMC13588049; doi:10.1002/advs.202514365)
Supplement: Supplementary file 1 [file ADVS-13-e14365-s001.docx]

*Supporting Information*

**Conformable Microelectrode Arrays Integrated with a Scoop-Shaped Slide-Well for Dynamic Electrophysiological Profiling of Patient-Derived Cardiac Organoids**

*Ye Seul Kim, Jeonghwa Jeong, Gyeonghwa Heo, Young Woo Kwon, Jeongha Lee, Yoon Ji Jeong, Gyu Tae Park, Seongdo Jeong, Ki Won Hwang, Murim Choi, Jinhong Shin, Suck Won Hong*, and Jae Ho Kim**

Dr. Y. S. Kim, Y. J. Jeong, Dr. G.T. Park, Prof. J. H. Kim

Department of Physiology

Department of Convergence Stem Cell Research Center

School of Medicine

Pusan National University

Yangsan 50612, Republic of Korea

E-mail: jhkimst@pusan.ac.kr (J.H.K.)

J. Jeong, G. Heo, Prof. S. W. Hong

Department of Optics and Mechatronics Engineering

Department of Cogno-Mechatronics Engineering

College of Nanoscience and Nanotechnology

Pusan National University

Busan 46241, Republic of Korea

E-mail: swhong@pusan.ac.kr (S.W.H.)

Dr. Y. W. Kwon

Engineering Research Center for Color-Modulated Extra-Sensory Perception Technology

Pusan National University

Busan 46241, Republic of Korea

J. Lee, Prof. M. Choi

Department of Biomedical Sciences

College of Medicine

Seoul National University

Seoul 03080, Republic of Korea

Dr. S. Jeong

Research Institute for Convergence of Biomedical Science and Technology

Pusan National University

Yangsan 50612, Republic of Korea

Prof. K.W. Hwang

Division of Cardiology

Department of internal medicine

School of medicine

Pusan National University Yangsan Hospital

Yangsan 50612, Republic of Korea

Prof. J. Shin

Department of Neurology

College of Medicine

Pusan National University Yangsan Hospital

Yangsan 50612, Republic of Korea

**Supplemental Notes**

**Supplemental Discussion**

In this study, we identified a frameshift mutation in exon 46 and a nonsense mutation in exon 60 of the gene in two DMD patients. This mutation leads to a truncation of the dystrophin domain responsible for interacting with the glycoprotein complex, thereby compromising the anchoring of the cytoskeleton to the ECM. It is well established that such structural uncoupling destabilizes the sarcolemma and disrupts ion channel function. Consistent with this mechanism, our DMD COs exhibited profound electrophysiological deficits, including reduced field potential amplitude and impaired calcium flux, which mirror the reduced sodium current density and contractility defects observed in *mdx* mice and other cardiomyopathy models.^[^^1^^]^ Furthermore, the morphological analysis revealed that DMD COs possessed significantly enlarged surface areas. This expansion likely recapitulates the structural remodeling associated with early-stage dilated cardiomyopathy, potentially driven by pathological hypertrophy or aberrant ECM accumulation indicative of fibrosis.^[^^1b^^]^ Critically, these structural and ionic maladaptations culminated in functional instability. While normal COs maintained stable rhythm, our DMD models frequently displayed arrhythmogenic firing patterns and oscillatory potentials. This aligns with previous findings in hiPSC-CMs where specific DMD mutations (e.g., exon 8–9 or 3–7 deletions) were linked to excessive intracellular calcium accumulation and delayed depolarization.^[^^2^^]^ Another mutation in the DMD gene was linked to increased mitochondrial calcium uptake in CMs, contributing to electrophysiological abnormalities and the onset of arrhythmias in DMD. In a DMD cardiomyopathy model, the combination of exon 8–9 deletions with an exon 3–7 deletion caused excessive intracellular calcium accumulation and further altered action potential dynamics.^[^^3^^]^ Collectively, these findings confirm that the DMD CO model faithfully reproduces both the structural remodeling and electrophysiological hallmarks of the disease, providing a physiologically relevant platform for dissecting the mechanisms of DMD cardiomyopathy.

While structural abnormalities such as large lumen formation were evident in our DMD organoids, interpreting the underlying cellular dynamics requires careful distinction between proliferation and remodeling. Previous studies have reported that DMD-derived CMs often exhibit reduced proliferative capacity due to diminished YAP signaling.^[^^4^^]^ However, in the present study, we demonstrated that DMD COs displayed a marked increase in overall size. Our transcriptomic profiling showed that the enlargement is likely not driven by hyperplasia, but by pathological ECM accumulation and compensatory hypertrophy. This is supported by the significant upregulation of fibrosis-associated genes (e.g., *COL1A2*, *COL3A1*, and *FN1*) and the emergence of adipogenic signatures,^[^^1b^^]^ confirming that the morphological expansion reflects the fibrotic progression inherent to dystrophin-deficient tissues rather than a simple aggregation artifact.

The coexistence of downregulated calcium and potassium channel expression with dynamic intracellular calcium transients suggests that the observed arrhythmias are driven by a mechanism of "sodium-driven calcium overload," distinct from primary L-type calcium channel flux. While voltage-gated calcium currents are reduced, our findings align with the model^[^^5^^]^**,** where an enhancement of the late sodium current (late I_Na_) acts as a critical upstream trigger for intracellular calcium instability. As established in heart failure models, elevated intracellular sodium([Na^+^]_i_) forces the Na^+^/Ca^2+^ exchanger (NCX) into reverse mode, importing calcium to extrude excess sodium. This secondary calcium entry is sufficient to overload the sarcoplasmic reticulum (SR), leading to spontaneous calcium release events known as dynamic transients. This mechanism is further exacerbated by the observed reduction in potassium channels; the resulting delay in repolarization extends the window for sodium and calcium entry, creating a vicious cycle of ionic accumulation and electrical instability. Although our COs model does not harbor primary genetic defects in the ryanodine receptor (RyR2), the observed calcium dynamics mimic the "leaky" RyR2 phenotype. Previous studies indicate that cellular calcium overload activates CaMKII, which in turn phosphorylates RyR2, increasing its open probability and sensitivity to luminal calcium.^[^^6^^]^ This PKA/CaMKII-mediated hyperphosphorylation dissociates the stabilizing subunit FKBP12.6 from the channel, rendering wild-type RyR2 functionally unstable.^[^^7^^]^ This acquired dysfunction leads to diastolic calcium leak, which generates transient inward currents (I_T1_) responsible for Delayed Afterdepolarizations (DADs) and sustained triggered activity.^[^^5^^]^ Thus, the "dynamic calcium transients" observed in our study likely represent DADs driven by the synergistic effects of APD prolongation (due to low K^+^) and functional RyR2 leak (due to Na^+^/Ca^2+^ overload). The presence of high collagen content indicates that these ionic disturbances have translated into structural remodeling. Sodium channel dysfunction and the resulting intracellular ion dysregulation can activate pro-fibrotic signaling pathways, such as TGF-b, thereby linking electrical defects to structural degeneration.^[^^8^^]^ Atrial fibrosis alone is sufficient to increase arrhythmia vulnerability by disrupting the electrical continuity of the myocardium and increasing conduction heterogeneity.^[^^9^^]^ Consequently, the high collagen content observed in our model likely stabilizes the re-entrant substrates necessary to maintain arrhythmias initiated by the focal triggers of calcium overload.^[^^10^^]^ This integrates our findings into a cohesive pathology where ionic remodeling drives secondary calcium instability and downstream fibrosis, creating a highly vulnerable substrate for sustained arrhythmia. Furthermore, while distinct morphological variations were observed (Figure S24, Supporting information), this likely reflects the phenotypic heterogeneity and structural disorganization characteristic of severe dystrophin deficiency, rather than experimental inconsistency. It is postulated that these defects are exacerbated by disorganized microtubule assembly, which further weakens contractile coordination. Collectively, our data suggest that genetic variations in DMD initiate widespread, organ-level disorganization during cardiogenesis. By mirroring these patient-specific pathologies, the DMD CO model serves as a powerful platform for elucidating the mechanisms of X-linked DMD cardiomyopathy and offers a robust tool for early disease detection, therapeutic screening, and clinical prognosis.

The uniform and conformal contact between the flexible MEA and the organoid surface in a fluidic environment was quantitatively verified by impedance measurements at 300 Hz (n = 16 electrodes). Upon organoid placement, electrode impedance decreased significantly and consistently from approximately 250 kΩ in medium alone to ~100 kΩ after attachment (paired t-test, p < 0.001), which is consistent with improved electrode–tissue coupling and a reduced intervening electrolyte layer, thereby minimizing contact-related artifacts (Figure S25, Supporting Information).^[^^11^^]^ Notably, post-attachment impedance values showed low electrode-to-electrode variability, indicating that conformal coupling was established across the array rather than being restricted to a subset of sites. In this regime, the absence of a statistically significant correlation between post-attachment impedance and signal parameters (amplitude: r = −0.16; SNR: r = −0.01) supports the interpretation that spatial heterogeneity in EFP waveforms, amplitudes, and onset latencies primarily reflects intrinsic biological variability rather than non-uniform coupling.^[^^12^^]^ This conclusion is further supported by two orthogonal lines of evidence: single-cell transcriptomics revealing heterogeneous ion-channel gene expression (Figure 3)^[^^13^^]^ and Fluo-4 AM calcium imaging demonstrating region-dependent calcium-handling profiles. Together, these data validate that inter-channel signal variations capture genuine, region-specific electrophysiological landscapes of COs enabled by the reliable and consistent interface of the deformable MEA platform.

**Comparative morphogenesis of normal and DMD COs**

Following the progression through mesodermal and cardiac progenitor stages, spontaneous beating was observed in normal COs. As shown in the representative images, no significant lumen formation or diameter change was evident in either group by day 2 of differentiation. However, by day 4, DMD COs exhibited a marked increase in size, and aberrant cavity-like structures began to form. Despite their increased size, DMD COs did not display synchronized contractions throughout the entire structure; instead, disorganized and localized beating was observed beginning around day 10.

In the context of human fetal development, cardiac beating typically initiates around day 22 of gestation, with heart tube looping occurring between days 22 and 28 (Table S4, Supporting Information). To compare the temporal dynamics of embryonic cardiac development and organoid morphogenesis, we examined organoids at 12, 18, 22, and 27 days post-differentiation (Figure S8, Supporting Information). In normal COs, linear heart tube-like structures became evident between days 14 and 17 (Figure S8a, Supporting Information). This was followed by dynamic changes in cell size over the next week, likely driven by the concurrent growth of cardiomyocytes and structural remodeling of the tissue. By day 22, tube looping and initial chamber formation became apparent (Figure S8b–c, Supporting Information). Morphologically, these emerging chamber-like structures were reminiscent of primitive outflow tract formation and early ventricular partitioning. However, fully developed chamber boundaries were not yet evident by day 27 (Figure S8d, Supporting Information).

In contrast to the organized architecture of normal COs, which displayed well-defined chamber-like structures populated by CMs and vascular networks (Figure S9a, Supporting Information), DMD COs exhibited impaired morphogenesis. Furthermore, while normal COs showed robust cTnT expression, DMD COs displayed disrupted cellular organization along with significantly diminished cTnT immunofluorescence signals (Figure S9b). To confirm the pathological phenotype, we conducted immunostaining for dystrophin using an antibody targeting its C-terminal domain. This approach is relevant because DMD-associated mutations result in a truncated dystrophin protein due to premature termination. As expected, dystrophin expression was markedly reduced in DMD COs compared to normal COs (Figure S9c, Supporting Information).

**Bulk RNA sequencing analysis in DMD CO**

We assessed the impact of the *DMD* mutation on gene expression patterns associated with cardiac development and pathology. By comparing the transcriptomic profiles of DMD and normal COs, we identified significant alterations in genes essential for cardiac structure and function (Figure S12a, Supporting Information). Gene Ontology (GO) analysis revealed that normal COs were significantly enriched in terms related to cardiac-specific biological processes, such as muscle system function, striated muscle cell differentiation, and structural components of the sarcomere and myofibrils. In contrast, DMD COs exhibited an aberrant upregulation of genes associated with non-cardiac lineages, including nephron-like epithelial and digestive system processes. This suggests that DMD COs display lineage infidelity or impaired fate determination, potentially reflecting ectopic differentiation toward endodermal or mesodermal derivatives at the expense of cardiac specification. Correlation analysis further demonstrated that transcriptomic profiles were highly consistent across independent iPSC clones within the 3D organoid platform, whereas they showed significant divergence when compared to 2D-cultured hiPSC-CMs (Figure S12b, Supporting Information). This highlights the profound impact of the 3D microenvironment on gene expression, distinguishing organoid models from conventional monolayer cultures regardless of disease background. To explore the functional implications of these alterations, we analyzed protein–protein interaction networks among the highly differentially expressed genes (Figure S12c, Supporting Information). We identified nine core genes governing critical aspects of cardiac physiology: muscle contraction and relaxation (*TNNI1, TRDN, ACTA1*), cardiac development and calcium handling (*NKX2-5, PLN*), and energy metabolism and structural stability (*ENO3, CKMT2, TRIM54, CMYA5*). The widespread downregulation of these genes in DMD COs aligns with the structural and functional defects observed in our model, recapitulating the hallmark features of DMD-associated cardiomyopathy, such as progressive myocardial weakening, dilation, and impaired contractility.

**Bulk RNA sequencing analysis about KEGG pathway in DMD CO**

Comparative analysis of differentially expressed genes (DEGs) across 2D and 3D platforms revealed that normal COs exhibited the robust expression of cardiac-specific genes, surpassing their 2D counterparts. However, DMD COs showed a significant downregulation of gene sets essential for proper cardiac development and maturation (Figure S12d, Supporting Information). To gain mechanistic insight, we performed pathway analysis using the Kyoto Encyclopedia of Genes and Genomes (KEGG) database. This revealed distinct biological divergences between normal and DMD COs. Notably, pathways governing "Cardiac muscle contraction" and "cAMP signaling", which are critical for excitation-contraction coupling and calcium handling, were significantly downregulated in DMD COs on day 25. This molecular deficit correlates with the impaired contractility and calcium handling defects observed in our functional assays. Conversely, non-cardiac pathways such as "Pancreatic secretion" and "Protein digestion and absorption" were aberrantly upregulated (Figure S12e, Supporting Information). This paradoxical activation of endoderm-associated metabolic pathways further corroborates the lineage infidelity and pathological remodeling characteristic of the dystrophic organoids.

**Transcriptomic profiling of cardiac lineage specification in DMD CO**

Through extended DEG analysis, we identified 29 core genes critically involved in cardiac metabolism and structure (Figure S13, Supporting Information). These genes were categorized based on their physiological roles: (1) muscle contraction and structural organization (e.g., *MYH7, TNNI3, MYBPC3*); (2) cytoskeletal stabilization (e.g., *LDB3, CSRP3*); (3) large myocyte-associated structural proteins (notably *TTN*); (4) calcium signaling and ion regulation (e.g., *RYR2, PLN*); and (5) energy metabolism (e.g., *CKM, MB*). Additionally, *RBM20* was identified as a key regulator of alternative splicing (Figure S13a,b). To explore the functional implications of these alterations, we analyzed a protein–protein interaction (PPI) network of these 29 core genes (Figure S13c). The network demonstrates a tightly interconnected topological structure, highlighting that the widespread downregulation observed in DMD COs collectively destabilizes a unified core network governing cardiac electromechanical function.

Comparative expression analysis revealed a low correlation between normal 2D-CMs and normal 3D-COs, whereas DMD COs retained high transcriptomic similarity to normal COs. This suggests that while the 3D microenvironment fundamentally shapes the global cardiac transcriptome distinct from 2D cultures, disease-specific perturbations occur within this preserved organoid identity. Despite this global similarity, DMD COs exhibited the specific upregulation of non-cardiac genes, including those associated with renal slit diaphragm components (*KIRREL2, NPHS1*) and digestive enzymes (*CPA1, CPA2*), confirming the presence of lineage infidelity (Figure S14, Supporting Information). These findings indicate that impaired gene regulatory networks in DMD COs lead to reduced cardiac specialization. Crucially, these transcriptional abnormalities provide the molecular basis for the overt cardiac dysfunctions observed in our functional assays, rather than merely reflecting early-stage deviations. This aligns with the hallmark pathological features of DMD, such as fibrosis and fatty infiltration, where functional myocardium is progressively replaced by non-cardiac tissues. Collectively, the transcriptomic profile of DMD COs underscores a compromised cardiac identity driven by disrupted gene expression programs and aberrant differentiation trajectories. Furthermore, we identified a critical disruption in lineage fidelity. At week 4, DMD COs showed aberrant expansion of PTF1A, a pancreatic progenitor marker. This suggests that the signaling landscape in DMD is sufficiently perturbed to allow leakage into non-cardiac lineages. We attribute this mis-specification to the dysregulation of WNT, BMP, and TGFβ signaling pathways, which are pivotal for cardiac commitment but are frequently altered in dystrophin-deficient models.^[^^14^^]^ Consequently, the upregulation of PTF1A indicates that the pro-fibrotic microenvironment may inadvertently create a niche that promotes phenotypic heterogeneity. By disrupting the delicate balance of developmental signals, the DMD pathology drives organoids toward structural disorganization and ectopic endodermal differentiation. These findings underscore the utility of our model in capturing the complex, multi-lineage consequences of dystrophin deficiency beyond simple muscle weakness.

**Enlarged ECM accumulation in DMD CO**

To validate the ECM accumulation suggested by the scRNA-seq analysis, we performed a comprehensive set of histological and biochemical assays, including Masson’s trichrome staining and hydroxyproline quantification (Figure S16a–c, Supporting Information). Although elevated collagen deposition is a hallmark of DMD, previous reports indicate that these increases may vary in significance depending on the tissue sample.^[^^15^^]^ Masson’s trichrome staining was performed on 5 μm cryo-sections of organoids at 4 weeks of differentiation. Consistent with the dense cellular aggregation observed, DMD COs displayed extensive blue staining throughout the tissue, indicating significant collagen deposition compared to the minimal staining in normal COs. To quantify this fibrosis, we measured hydroxyproline content. While levels were comparable between groups at the early stage (2 weeks, Figure S16b), DMD COs exhibited a marked increase in hydroxyproline concentration by 4 weeks (Figure S16c). Interestingly, quantitative analysis of the cardiac fibroblast population revealed a reduction in DMD COs compared to normal COs, despite the increased fibrotic burden (Figure S16d,e, Supporting Information). This inverse relationship suggests that the observed fibrosis is likely driven by the pathological hyperactivity of the remaining fibroblasts or the profibrotic transition of cardiomyocytes, as indicated by our transcriptomic data, rather than fibroblast proliferation alone. These findings confirm that DMD COs recapitulate the delayed maturation and extensive ECM remodeling characteristics of dystrophic cardiomyopathy.

**Characterization of fibroblast subtypes in DMD CO**

Cardiac fibroblasts, which comprise a major cellular component of the heart, play a pivotal role in maintaining three-dimensional tissue architecture by regulating the turnover of extracellular matrix (ECM) proteins.^[^^16^^]^ Typically, in response to myocardial damage, the deposition of collagen and other matrix proteins increases as part of the reparative fibrosis process.^[^^17^^]^ Interestingly, while collagen accumulation was evident, DMD COs in our study exhibited a reduced expression of fibronectin, a key adhesive ECM glycoprotein, compared to normal COs. This suggests a specific dysregulation of ECM composition rather than a uniform upregulation of all matrix components. Previous reports indicate that DMD pathology involves the chronic activation of myofibroblasts and a disrupted balance between collagen synthesis and degradation by matrix metalloproteinases (MMPs), ultimately driving interstitial fibrosis.^[^^18^^]^

Beyond the cardiomyocyte population, scRNA-seq analysis resolved several distinct fibroblast subtypes within the COs. These subpopulations included Neuromuscular, Myofibroblast, Cycling, and Inflammatory fibroblasts, alongside distinct mesenchymal clusters characterized by the differential expression of *LUM* (*LUM^low/high^*) and *DCN/MYC* (*DCN^low^/MYC^high^* vs. *DCN^high^/MYC^low^*) (Figure S17a, Supporting Information). The top five marker genes defining each fibroblast cluster are detailed in Figure S17b. We also noted donor-specific variations in the proportional composition of these subsets (Figure S17c).

To elucidate the pathological mechanisms driving fibrosis, we performed DEG analysis specifically targeting the pathologically active myofibroblast subset. This analysis revealed a significant transcriptional shift in DMD myofibroblasts (Figure S17d,e, Supporting Information): genes associated with muscle contraction and development were notably downregulated, whereas those encoding structural ECM components were consistently upregulated. This shift indicates that DMD myofibroblasts acquire a hyper-synthetic phenotype, prioritizing matrix secretion over contractile or developmental functions.

**Immunofluorescence analysis of cardiac lineage specificity in DMD CO**

To evaluate the spatial specificity of cardiac differentiation, we performed immunofluorescence analysis targeting lineage-specific markers. Both normal and DMD organoids exhibited distinct spatial segregation of the atrial region (marked by the atrial marker MLC2a); however, the expression of the ventricular marker MLC2v remained notably weak in DMD organoids. In normal COs, phalloidin staining revealed the formation of organized tubular architectures representing robust cytoskeletal integrity, which were spatially distinct from the atrial domains. In contrast, DMD organoids displayed limited and patchy expression of ventricular lineage markers, accompanied by disorganized actin filaments. These findings indicate that while atrial specification may initiate, DMD organoids exhibit a specifically delayed or impaired ventricular lineage development (Figure S19, Supporting Information).

**Supplemental methods**

**Whole exome sequencing (WES) analysis:** Table S3 details the pathogenic variants identified via WES. While several incidental pathogenic variants, including those in the *ABCD1* and *IGSF3* genes, were observed even in the normal group, these are not known to be involved in cardiac development or function. In the DMD samples, variants in *IGSF3* were identified alongside the expected DMD gene mutations. Crucially, to date, no direct correlation or functional link has been reported between *IGSF3* and cardiac morphogenesis. Therefore, we infer that these background variants function as independent variables and do not act as confounding factors contributing to the cardiac phenotypes observed in this study.

**Bulk RNA sequencing analysis:** Total RNA was extracted from normal and DMD COs. Library preparation, high-throughput sequencing, and initial bioinformatic analyses were performed by DNA Link (Republic of Korea). Sequencing libraries were prepared using the TruSeq Stranded mRNA Library Prep Kit (Illumina, San Diego, CA, USA) according to the manufacturer’s instructions. High-throughput sequencing was performed on the Illumina NovaSeq platform. Base calling and demultiplexing of raw image data were carried out using bcl2fastq2 software (version 2.20, Illumina), generating raw sequence data in FASTQ format with an ASCII Q-score offset of 33. Transcript abundance was quantified and pseudoalignment to the human reference transcriptome was performed using kallisto. Differential expression analysis was subsequently conducted using the edgeR package in R. Significantly differentially expressed genes (DEGs) were defined as those with an absolute log2 fold change ≥ 1 and an adjusted p-value ≤ 0.05. Statistical significance for gene ontology (GO) and KEGG enrichment analyses, performed using clusterProfiler, as well as for gene set enrichment analysis (GSEA), was assessed using adjusted p-values. Across all transcriptomic analyses, adjusted p-values were calculated using the Benjamini-Hochberg method to control the false discovery rate (FDR), and an FDR ≤ 0.05 was considered statistically significant.

**Functional enrichment and protein–protein interaction (PPI) analysis:** DEGs were further analyzed through GO and KEGG enrichment analyses to identify significantly altered biological pathways. These analyses were performed using the clusterProfiler package (v4.0.5) in R. To control the FDR, adjusted p-values were calculated using the Benjamini-Hochberg method, and enriched terms with an adjusted p-value ≤ 0.05 were considered statistically significant. In addition, PPI networks were constructed to investigate functional associations among the core DEGs using the STRING database, with a minimum required interaction score of 0.400 (medium confidence).

**Detailed Methods for Statistical Analysis:** Data preprocessing included normalization of the relevant experimental measurements. Western blot band intensities were normalized to GAPDH, qPCR data were normalized to a housekeeping gene, and scRNA-seq gene expression counts were normalized using the LogNormalize method. Prior to statistical testing, datasets were screened for outliers, which were identified and excluded using the interquartile range (1.5 × IQR) method. For scRNA-seq analysis, the sample size (*n*) refers to the number of independent biological replicates rather than the number of individual cells. Specifically, *n* = 2 biological replicates were analyzed per group (Normal and DMD). Each biological replicate consisted of a pooled sample of 48 individual cardiac organoids. From each pooled sample, we targeted a recovery of approximately 20000 cells per run. Differential gene expression (DEG) analysis and subsequent statistical comparisons were performed at the level of biological replicates to avoid pseudo-replication. Significant DEGs were defined as those with an adjusted *p*-value ≤ 0.05 and an absolute log2 fold change ≥ 1. The statistical significance of GO term enrichment was assessed using adjusted *p*-values calculated by the Benjamini-Hochberg method. Relationships among gene expression profiles were evaluated using Pearson correlation analysis. For standard quantitative comparisons, a paired two-tailed Student’s t-test was used for matched measurements (e.g., pre- and post-contact impedance). For comparisons between two independent groups that did not satisfy the normality assumption, the two-tailed nonparametric Mann-Whitney test was used. For comparisons involving more than two groups that satisfied the normality assumption, one-way ANOVA followed by Tukey’s post hoc multiple-comparison test, or two-way ANOVA followed by Sidak’s multiple-comparisons test, was applied as appropriate for the experimental design. Non-significant comparisons were either omitted from the graphs for clarity or explicitly indicated as “n.s.”.

**Table S1**. Sequence analysis of the *DMD* gene in two DMD patients.

| **Gene** | **Variant Coordinates** | **In silico parameters** | **Allele Frequencies** | **Type and Classification** |
| --- | --- | --- | --- | --- |
| DMD1 | c.6655_6656delGA(p.D2219Ffs*3) | MutationTaster: deleterious | Not found in ExAC, 1000G, or gnomAD | Frameshift  Pathogenic (PVS1, PM2, PP4) |
| DMD2 | c.8944C>T  (p.R2982*) | MutationTaster: deleterious | Not found in ExAC, 1000G, or gnomAD | Nonsense  Known pathogenic (PVS1, PP4, PP5) |

**Table S2**. Analysis of the DMD gene in four iPSCs in whole exome sequencing

| **Group** | **No.** | **Gene** | **Variant** | | **Impact/**  **Clinical significance** |
| --- | --- | --- | --- | --- | --- |
| **Normal** | 1 | DMD | Missense | c.8810G>A | Moderate/Benign |
|  |  |  | Missense | c.5234G>A | Moderate/Benign |
|  |  |  | Missense | c.2645A>G | Moderate/Benign |
| **Normal** | 2 | DMD | Synonymous | c.7728T>C | Low/ N.A |
|  |  |  | Intron | c.2168+13T>C | Modifier/ N.A. |
|  |  |  | Missense | c.5163G>C | Moderate/ Benign&Likely_benign |
|  |  |  | Missense | c.8810G>A | Moderate/ Benign |
| **DMD** | 1 | DMD | Frameshift | c.6655_6656delGA | High/N.A. |
|  |  |  | Stop_gained & conservative inframe_insertion | c6136_6137insGGT | High/N.A. |
|  |  |  | Missense | c.8810G>A | Moderate/Benign |
|  |  |  | Missense | c.5234G>A | Moderate/Benign |
|  |  |  | Missense | c.2645A>G | Moderate/Benign |
| **DMD** | 2 | DMD | Stop_gained | c.8944C>T | High/ Pathogenic |
|  |  |  | Missense variant | c.8810G>A | Moderate/Benign |
|  |  |  | Missense variant | c.5234G>A | Moderate/Benign |
|  |  |  | Missense variant | c.2645A>G | Moderate/Benign |

**Table S3**. Analysis of the pathogenic genes in four iPSCs in whole exome sequencing

| **Group** | **No.** | **Chrom.** | **Gene** | **Varient** | | **Impact** | **Clinical significance** |
| --- | --- | --- | --- | --- | --- | --- | --- |
| **Normal** | **1** | **chrX** | *ABCD1* | stop_gained | c.1699C>T | HIGH | Pathogenic |
|  |  | **chr1** | *IGSF3* | stop_gained | c.1784G>A | HIGH | Pathogenic |
|  |  | **chrX** | *SERPINA7* | missense | c.909G>T | MODERATE | Pathogenic |
|  |  | **chrX** | *ABCD1* | missense | c.1661G>A | MODERATE | Pathogenic |
|  |  | **chr7** | *CD36* | missense | c.268C>T | MODERATE | Pathogenic |
|  |  | **chr6** | *CYRP21A2* | missense | c.518T>A | MODERATE | Pathogenic |
|  |  | **chr5** | *FGFR4* | missense | c.1162G>A | MODERATE | Pathogenic |
|  |  | **chr11** | *PTPRJ* | missense | c.827A>C | MODERATE | Pathogenic |
|  |  | **chr10** | *MBL2* | missense | c.161G>A | MODERATE | Pathogenic |
| **Normal** | **2** | **chr4** | *COQ2* | missense | c.382A>G | MODERATE | Pathogenic,_risk_factor |
|  |  | **chr3** | *GHRL* | missense | c.214C>A | MODERATE | Pathogenic,_risk_factor |
|  |  | **chr3** | *GHRLOS* | intron | n.566+1667G>T | MODIFIER | Pathogenic,_risk_factor |
|  |  | **chr11** | *PTPRJ* | missense | c.827A>C | MODERATE | Pathogenic |
|  |  | **chr10** | *PRKCQ* | missense | c.989C>T | MODERATE | Pathogenic |
|  |  | **chr1** | *IGSF3* | stop_gained | c.1784G>A | HIGH | Pathogenic |
| **DMD** | **1** | **chr6** | PLA2G7 | missense | c.835G>T | MODERATE | Pathogenic,_risk_factor |
|  |  | **chr16** | IL4R | missense | c.223A>G | MODERATE | Pathogenic,_protective |
|  |  | **chrX** | ABCD1 | stop_gained | c.1699C>T | HIGH | Pathogenic |
|  |  | **chrX** | ABCD1 | missense | c.1661G>A | MODERATE | Pathogenic |
|  |  | **chr5** | FGFR4 | missense | c.1162G>A | MODERATE | Pathogenic |
|  |  | **chr1** | IGSF3 | stop_gained | c.1784G>A | HIGH | Pathogenic |
| **DMD** | **2** | **chr6** | PLA2G7 | missense | c.835G>T | MODERATE | Pathogenic,_risk_factor |
|  |  | **chr3** | GHRL | missense_ | c.214C>A | MODERATE | Pathogenic,_risk_factor |
|  |  | **chr3** | GHRLOS | intron | n.566+1667G>T | MODIFIER | Pathogenic,_risk_factor |
|  |  | **chr16** | IL4R | missense | c.223A>G | MODERATE | Pathogenic,_protective |
|  |  | **chrX** | DMD | stop_gained | c.8944C>T | HIGH | Pathogenic |
|  |  | **chr5** | FGFR4 | missense | c.1162G>A | MODERATE | Pathogenic |
|  |  | **chr10** | PRKCQ | missense | c.989C>T | MODERATE | Pathogenic |
|  |  | **chr1** | IGSF3 | stop_gained | c.1784G>A | HIGH | Pathogenic |

**Table S4**. The development timeline of human heart embryology spans approximately 57 days from fertilization to the completion of ventricular septation, with each key developmental stage distinctly defined throughout this process. Reproduced with permission from University of Minnesota.^[^^19^^]^

| **Human Development (days)** | **Developmental process** |
| --- | --- |
| 0 | Fertilization |
| 1-4 | Cleavage and movement down the oviduct to the uterus |
| 5-12 | Implantation of the embryo into the uterus |
| 13-14 | Primitive streak formation (midstreak level contains precardiac cells) |
| 15-17 | Formation of the three primary germ layers (gastrulation): ectoderm, mesoderm, endoderm;  Midlevel primitive streak cells that migrate to an anterior and lateral position form the first heart field and more medially the second heart field |
| 17-18 | Lateral plate mesoderm splits into somatopleuric and mesoderm and splanchnopleuric mesoderm;  Splanchnopleuric mesoderm contains the myocardial and endocardial cardiogenic precursors in the region of the first heart field and second heart field |
| 18-26 | Neurulation (formation of the neural tube) |
| 20 | Cephalocaudal and lateral folding brings the bilateral endocardial tubes into the ventral midline of the embryo |
| 21-22 | Heart tube fusion |
| 22 | Heart begins to beat |
| 22-28 | Heart looping and accretion of cells from the first and second heart fields;  Proepicardial cells invest from the outer layer of the heart tube and eventually form the epicardium and coronary vasculature;  Neural crest migration starts |
| 32-37 | Cardiac neural crest migrates through the aortic arches and enters the outflow tract of the heart |
| 57+ | Outflow tract and ventricular septation complete |
| Birth | Functional septation of the atrial chambers, as well as the pulmonary and systemic circulatory systems |

**Table S5**. Ratio of distribution of organoid subtypes at Figure 3b

| **Cluster** | **DMD1** | **DMD2** | **Normal1** | **Normal2** | **DMD** | **Normal** |
| --- | --- | --- | --- | --- | --- | --- |
| Hepatic/Pancreatic_progenitor | 0.0388 | 0.907 | 0.048 | 0.00661 | 0.945 | 0.0546 |
| Cycling_hepa/pancreatic | 0.0538 | 0.692 | 0.111 | 0.143 | 0.746 | 0.254 |
| Hepatocyte | 0.114 | 0.232 | 0.209 | 0.446 | 0.346 | 0.654 |
| cholangiocyte | 0.104 | 0.242 | 0.209 | 0.445 | 0.346 | 0.654 |
| vCM | 0.301 | 0.31 | 0.138 | 0.251 | 0.611 | 0.389 |
| LowQual_CM | 0.204 | 0.251 | 0.302 | 0.244 | 0.454 | 0.546 |
| sinoatrialCM | 0.0936 | 0.369 | 0.12 | 0.417 | 0.463 | 0.537 |
| fibrotic_CM | 0.237 | 0.185 | 0.379 | 0.199 | 0.422 | 0.578 |
| inflamedCM | NULL | 0.0101 | 0.985 | 0.00504 | 0.0101 | 0.99 |
| Cycling_CM | 0.272 | 0.279 | 0.171 | 0.278 | 0.551 | 0.449 |
| SignalRich_CM | 0.904 | 0.0342 | 0.0462 | 0.0154 | 0.938 | 0.0615 |
| Neuro-mascular | 0.133 | 0.227 | 0.0847 | 0.555 | 0.36 | 0.64 |
| myofibroblast | 0.08 | 0.394 | 0.148 | 0.379 | 0.474 | 0.526 |
| inflam_FB | NULL | 0.0117 | 0.986 | 0.00234 | 0.0117 | 0.988 |
| cycling_FB | 0.165 | 0.196 | 0.322 | 0.318 | 0.36 | 0.64 |
| DCN_low_MYC_high | 0.0109 | 0.00693 | 0.976 | 0.00594 | 0.0178 | 0.982 |
| LUM_low_mesen | 0.254 | 0.245 | 0.325 | 0.177 | 0.499 | 0.501 |
| LUM_high_mesen | 0.971 | NULL | 0.0294 | NULL | 0.971 | 0.0294 |
| Endothelium | 0.179 | 0.503 | 0.189 | 0.128 | 0.682 | 0.318 |
| Neurogenic_progenitor | 0.992 | 0.000657 | 0.00657 | 0.000657 | 0.993 | 0.00723 |
| RG/Glial_like | 0.928 | 0.067 | 0.0049 | NULL | 0.995 | 0.0049 |
| immature_neuron | 0.951 | 0.031 | 0.0181 | NULL | 0.982 | 0.0181 |
| Cycling_neuronal | 0.984 | 0.0157 | NULL | NULL | 1 | 0 |
| neuroendocrine | 0.0946 | 0.784 | 0.0541 | 0.0676 | 0.878 | 0.122 |

**Figure S1.** Impedance spectrum of Au and PEDOT:PSS-coated Au electrodes over the frequency range of 0.5–1,000 Hz, measured in phosphate-buffered saline (PBS) using an Ag/AgCl reference electrode and a Pt counter electrode.

**Figure S2.** Fabrication of the ultrathin MEA and PDMS scoop slide-well. a) Process flow for the ultrathin MEA: i) spin-coat and pattern SU-8 on a Si/SiO_2_ substrate, ii) deposit and pattern Cr/Au film to form microelectrodes, iii) encapsulate with a second SU-8 layer defining electrode windows, and iv) electrodeposit PEDOT:PSS on the exposed microelectrodes to tune impedance. b) Fabrication of the PDMS scoop-shaped slide-well: casting PDMS in a 3D-printed mold to yield a concave well, then combining the freestanding SU-8 MEA onto its curved surface.

**Figure S3**. Integration of the ultrathin MEA on a PDMS scoop-slide well. Digital and optical micrographs show the prototype platform in which the ultrathin MEA conformally adheres to the concave PDMS surface (upper panels). Side-view of the integrated MEA within the slide-well with microfluidic end, with a close-up of a CO trapped centrally on the electrode array (lower panels).

**Figure S4.** Impact of the signal preprocessing pipeline. a) Representative raw signal traces, b) The same traces after applying the preprocessing pipeline (60 Hz notch filter and DWT denoising.

**Figure S5**. Electrocardiograms of two patients with DMD. Top) ECG of DMD patient 1 showing a pattern consistent with early repolarization pattern; no overt arrhythmias was observed. Bottom) Abnormal ECG of DMD patient 2 indicating signs of both right and left ventricular hypertrophy.

**Figure S6.** Generation and validation of normal and DMD patient iPSCs. a) Representative bright-field images of urine-derived epithelial cells and the established iPSC colonies from healthy and DMD donors (scale bar = 500 µm). b) Immunofluorescence staining for the pluripotency markers SOX2 (green) and NANOG (red); nuclei counterstained with DAPI (blue). The images confirm the expression of key pluripotent factors in the reprogrammed cells. Scale bar = 100 µm. c) G-band karyotypes confirming normal chromosomal integrity in both lines.

**Figure S7.** Temporal morphological tracking and size quantification of normal and DMD COs during differentiation. Representative bright-field time-course images of organoids derived from normal (upper panels) and DMD (lower panels) iPSCs. COs were generated from EBs initially formed from 2000 iPSCs. Images span from Day 0 to Day 31, illustrating the progression of cardiac formation and structural development. Quantitative analysis of organoid size (area or diameter) over time is shown in the corresponding graphs. Scale bar = 1000 µm. Data are presented as mean ± SD. Sample sizes ranged from *n* = 30 to 66 for DMD COs and *n* = 19 to 74 for normal COs. Outliers were identified and removed using the IQR method (1.5 × IQR rule). Statistical significance was determined using a two-way ANOVA followed by Sidak’s multiple comparisons test. ^*^*p* < 0.05; ^$^*p* < 0.01; ^#^*p* < 0.001.

**Figure S8.** Sequential morphological development of normal COs during 27 days of differentiation. Representative bright-field images tracking the developmental stages of organoids generated from normal hiPSC lines. Embryoid bodies (EBs) were formed using two initial seeding densities: 2,000 cells/EB (top row of each panel) and 4000 cells/EB (bottom row). a) Onset of spontaneous beating and initial structural elongation. b) Heart tube elongation and looping: Organoids display extended, curved architectures recapitulating the linear heart tube stage. c) Advanced looping and initial chamber specification: Complex 3D morphologies emerge, suggestive of early cardiac remodeling. d) Chamber formation: Distinct chamber-like structures become defined, resembling the compartmentalization of the embryonic heart. *n* = 40 for each group. Scale bar = 500 µm.

**Figure S9.** Quantitative characterization of cardiac and vascularization in normal and DMD COs. a) Representative immunofluorescence images stained for nuclei (DAPI, blue), endothelial cells (CD31, red), and CMs (cTnT, green). The corresponding line-scan profiles display relative fluorescence intensities across the indicated regions, illustrating the spatial organization of vascular and cardiac markers. Scale bar = 200 µm. b) Quantification of cTnT fluorescence intensity normalized to DAPI signal area. Data confirm a significant reduction in cardiomyocyte abundance in DMD COs. Data are presented as mean ± SD; *n* = 8. Significance was calculated by the non-parametric Mann-Whitney test (^***^*p* < 0.001). c) Representative immunofluorescence images of cryosectioned normal and DMD COs stained for F-actin (Phalloidin, grey), α-sarcomeric actinin (αSA, green), and dystrophin (red). Nuclei were counterstained with DAPI (blue). All of the experiments are performed at least three times. Scale bar = 200 µm.

**Figure S10.** Quantification of Western blot analysis in Figure 2e. Quantification of Western blot analysis comparing the expression levels of structural (Dystrophin, cTnT, ZO1, and MLC2a versus MLC2v ratio), ion channel (CaV1.2, HCN4, SERCA2a), and gap junction (CX43) proteins. Protein levels were normalized to GAPDH. Data are presented as mean ± SD (*n* = 4-6, independent experiments). Statistical significance was calculated using the non-parametric Mann-Whitney test (^*^*p* < 0.05; ^**^*p* < 0.01; ns, not significant).

**Figure S11.** Temporal protein expression profiles of cardiac lineage-specific markers in normal and DMD COs. Representative blots and quantification of atrial (MLC2a), ventricular (MLC2v), and pacemaker (HCN4) at Day 21 and Day 31. Each lane represents an independent biological replicate derived from a separate pool of organoids. The relative protein expression level of HCN4 and the ratio of MLC2a vs. MLC2v are shown in the right panels. All protein band intensities were normalized to GAPDH. Data are presented as mean ± SD (*n* = 5, biologically independent pools for normal COs; *n* = 4, biologically independent pools for DMD COs). Statistical significance was determined using the non-parametric Mann-Whitney test (^**^*p* < 0.01 and ^***^*p* < 0.001).

**Figure S12.** Transcriptomic analysis of cardiac dysfunction in DMD COs reveals dysregulated cardiac lineage specification and contractile gene networks. a) Gene ontology enrichment analysis showing significantly downregulated biological processes in DMD COs, including sarcomere organization and muscle system processes. The color gradient represents the adjusted *p*-value, calculated using the Benjamini-Hochberg method for multiple testing correction. b) Correlation matrix comparing gene expression patterns across normal and DMD COs and 2D-cultured CMs, highlighting distinct transcriptional profiles of 3D organoid models. The color scale ranges from -1.0 to 1.0, representing the Pearson correlation coefficient. c) Protein–protein interaction network of differentially expressed cardiac regulatory genes in DMD COs, including *ACTA1, TNNI1, CMYA5, PLN,* and *NKX2-5*. Nodes represent proteins and edges indicate interactions based on the STRING database with a minimum required interaction score of 0.400 (medium confidence). The line color indicates the type of interaction evidence. d) KEGG pathway analysis indicating downregulation of cardiac muscle contraction and cAMP signaling, alongside upregulation of digestive and renal differentiation pathways in DMD COs. Differentially expressed genes were defined based on an |log2 fold change| ≥ 1 and an adjusted *p*-value ≤ 0.05. e) Additional gene ontology enrichment validating suppression of cardiac contractility and developmental programs in DMD COs. The color gradient represents the p. adjust, calculated using the Benjamini-Hochberg method for multiple testing correction. For the ontology enrichment analysis, differentially expressed genes were strictly selected based on > 2-fold change in expression (|log2 fold-change| ≥ 1) and FDR ≤ 0.05.

**Figure S13.** Comparative transcriptomic profiling of cardiac-specific genes in normal CM, organoid and DMD COs. a) Heatmap comparison of the expression levels of 27 key cardiac specific genes across the groups. Differentially expressed genes were defined based on an |log2 fold change| ≥ 1 and an adjusted *p*-value ≤ 0.05, calculated using the Benjamini-Hochberg method for multiple testing correction. b) Pearson correlation matrix analyzing the relationship of gene expression profiles within the cardiac structure and function module. The color scale ranges from -1.0 to 1.0, representing the Pearson correlation coefficient. c) PPI network of cardiac structure and function module genes was visualized using the STRING database with a minimum required interaction score of 0.400 (medium confidence). Nodes represent proteins, and edges indicate known or predicted interactions, with line color representing the source of interaction evidence.

**Figure S14.** Transcriptomic analysis and network characterization of ectopic lineage signatures in DMD COs. a) Heatmap showing gene expression patterns in nephron modules from normal and DMD CO. Differentially expressed genes were defined based on FDR ≤ 0.05 and |log2 fold change| ≥ 1, calculated using the Benjamini-Hochberg method for multiple testing correction. The color scale represents log2 fold change. Expression values are normalized. Red indicates upregulation and blue indicates downregulation. b) PPI network of nephron module genes visualized using the STRING database with a minimum required interaction score of 0.400 (medium confidence). Nodes represent proteins, and edges indicate known or predicted interactions, with line color representing interaction type. c) Pearson correlation matrix of Nephron module gene expression. The color scale indicates the Pearson correlation coefficient (-1.0 to 1.0). d) Heatmap of gene expression in foregut module from normal and DMD CO. Differentially expressed genes were defined based on FDR ≤ 0.05 and |log2 fold change| ≥ 1, calculated using the Benjamini-Hochberg method for multiple testing correction. The color scale represents log2 fold change. e) STRING PPI network of foregut module genes, constructed with a minimum required interaction score of 0.400 (medium confidence). Nodes represent proteins, and edges indicate known or predicted interactions, with line color representing interaction type. f) Pearson correlation matrix of foregut module genes. The color scale indicates the Pearson correlation coefficient (-1.0 to 1.0).

**Figure S15**. Single-cell transcriptomic landscape and functional pathway analysis. a) UMAP visualization of single-cell RNA sequencing data. Color-coded clusters identify distinct cell populations, including various CM subtypes (ventricular, sinoatrial, fibrotic etc.), fibroblasts, and other lineages such as hepatic, neuronal, and endothelial cells. b) Heatmap showing Normalized Enrichment Scores (NES) from GSEA across identified cell types. Pathway enrichment analysis was conducted with fgsea on MSigDB gene sets to define the biological identity of each cluster. Significant enrichment is denoted by asterisks (^*^*p* < 0.05). Key pathways related to cardiac muscle contraction, calcium signaling pathway, and ECM regulation are highlighted.

**Figure S16**. Histological and molecular characterization of CMs, fibroblasts, and ECM in normal and DMD COs. a) Representative Masson staining images of normal and DMD COs sections showing tissue morphology. *n* = 31 for normal CO; *n* = 13 for DMD COs; Scale bar = 500 µm. Quantification of collagen secretion via hydroxyproline assay at 2 weeks b) and 4 weeks c), highlighting differences between groups. *n* = 3-9. Data are presented as mean ± SD. Significance was determined by non-parametric Mann-Whitney test (n.s., non-significant; ^***^*p* < 0.001). d) Immunofluorescence staining of COs for nuclei (DAPI, blue), CMs (cTnT, green), and alpha smooth muscle actin (aSMA, red). Scale bars = 100 µm. e) Western blot analysis of markers specific to cardiac fibroblasts and the vascular system.

**Figure S17**. Single-cell transcriptomic resolution of fibroblast heterogeneity and myofibroblast remodeling in normal and DMD COs. a) UMAP embedding showing the subclustering of cardiac fibroblasts and mesenchymal cells. Distinct subtypes were identified, including myofibroblasts (MyoFB), cycling fibroblasts (Cycling_FB), inflammatory fibroblasts (Inflamed_FB), neuromuscular cells, LUM-low and LUM-high mesenchymal cells, and a DCN(low) MYC(high) population. b) Heatmap showing the top marker genes defining each cluster. c) Stacked bar plot showing the proportions of these subtypes in normal and DMD COs (*n* = 2 per group). d) Volcano plot illustrating differentially expressed genes (DEGs) in the MyoFB subpopulation between DMD and normal groups. Significant DEGs were defined as those with an absolute log2 fold change ≥ 1 and an adjusted *p*-value ≤ 0.05, calculated using the Benjamini-Hochberg method for multiple-testing correction. e) GO enrichment analysis of MyoFB-specific DEGs. Significantly enriched GO terms were defined by an adjusted p-value ≤ 0.05, calculated using the Benjamini-Hochberg method for multiple-testing correction. Upregulated genes in DMD myofibroblasts were associated with extracellular matrix structural constituents, whereas downregulated genes were related to muscle contraction. BP, biological process; CC, cellular component; MF, molecular function.

**Figure S18**. Single-cell transcriptomic profiling of atrial, ventricular, and sinoatrial CM subtypes. a) Feature plots validating the classification of distinct CM subtypes. b) Feature plots showing the expression patterns of ventricular-specific markers, including *MYH7*, *MYL2*, *IRX4*, and *HEY2.* c) Atrial populations characterized by the expression of *MYH6*, *MYL7*, *NPPA*, *NR2F2*, and *KCNJ3*. d) Sinoatrial subtype populations identified by *SHOX2*, *RGS6*, *NRXN3*, *HCN4*, and *TBX3* expression patterns.

**Figure S19.** Spatial organization and chamber-like patterning in normal- and DMD COs. Representative immunofluorescence images of normal and DMD COs reveal spatially distinct localization of atrial and ventricular regions, indicative of cardiac chamber specification. Higher-magnification images (yellow-boxed regions) reveal tubular structures with surrounding CMs, suggesting the emergence of internal lumen-like morphologies, potentially associated with vascular or neural crest-derived patterning. Scale bars = 200 μm (overview), 50 μm (magnified).

**Figure S20.** Expression patterns of gap junction proteins in CM subpopulations. Transcriptomic profiling of *GJA1*, *GJA5*, and *GJC1* visualized by violin plots and feature plots. The plots highlight the differential expression of these connexin isoforms across the identified CM subtypes.

**Figure S21**. Ion channel expression profiles and calcium handling properties in normal and DMD COs. a) Dot plot visualizing the expression patterns of major cardiac ion channel genes across all identified cell clusters. Dot size represents the percentage of cells expressing the gene, and color intensity indicates average expression levels. b) Heatmap displaying the expression of key calcium handling-related ion channels (*SLC8A1*, *RYR2*, *CACNB2*, and *CACNA1C*) specifically within CM subpopulations. c) Calcium transient analysis in normal and DMD COs. Representative calcium traces (top) and kymographs (bottom) are shown. Bar graphs quantify beat period, spike amplitude (F1/F0), calcium decay time, and spike time. Data are presented as mean ± SD (*n* = 6 biological replicates). Statistical significance was determined using the non-parametric Mann-Whitney test (^*^*p* < 0.05, ns: not significant).

**Figure S22.** Electrophysiological assessment and beat stability analysis of CMs with 100nM E-4031. a) Representative mean of field potential duration waveforms recorded from multiple. Black vertical lines indicate detected beat events. Identical signal displayed using narrow and wide voltage ranges. b) Beat period stability over time for four representative wells. Green points indicate stable beats, and red points indicate unstable, or outlier beats based on defined threshold. c) Representative voltage traces from stable beats in b), showing consistent inter-beat intervals and waveform morphology (green). d) Representative voltage traces from unstable beats in figure S22b, showing irregularities in beat intervals or waveform shape (red). Quantification of electrophysiological parameters, including beat period variability e), spike amplitude f), and spike slope g). Note that spikes were quantified using an adaptive threshold-based detection algorithm, which accurately captures low-amplitude signals even when they appear flattened on ± 10 mV scale. Gray bars represent group means; Individual points represent biological replicates. Data are presented as mean ± SD (*n* = 32 biological replicates). Statistical significance was determined using the Tukey's multiple comparison test in one-way ANOVA (^*^*p* < 0.05; ^**^*p* < 0.01; ^****^*p* < 0.0001; ns, not significant).

**Figure S23.** Expression patterns of KCNH2 in CM subpopulations between normal and DMD cardiac subtypes. a) Transcriptomic profiling of KCNH2 visualized by violin plots and feature plots. b) Dot plots highlight the differential expression of KCNH2 gene across DMD and normal cardiac subtypes.

**Figure S24.** Representative brightfield images of abnormal morphologies observed during organoid differentiation. Each panel displays two organoids under the same conditions, highlighting the different phenotypes observed during the culture process. a) Intracellular necrosis during cardiac organoid differentiation: organoids exhibit signs of internal cell death, with darkened regions and disrupted internal architecture. b) shows formation of a Yolk Sac-like structure during differentiation, with organoids not developing into cardiac cells that indicate uneven cell proliferation or structural instability at day 8–10. c) Cardiac organoid teratogenicity by cell sacs: irregular sac-like structures form on the surface of the organoids, suggesting teratogenic effects or ectopic differentiation at day 10–12. d) Abnormal heart tube growth–organoids exhibit distorted elongation and fail to mimic normal heart tube structures. e) Abnormal cell splitting in culture: organoids show fragmentation and unusual cellular separation, which impairs cohesive growth. f) Impaired differentiation due to fibrous material in the culture medium; the presence of fibrous or extracellular material appears to interfere with the differentiation and morphology of the organoids. Scale bar = 500 µm.

**Figure S25**. Verification of MEA–organoid coupling under fluidic recording conditions. Impedance magnitude at 300 Hz measured in culture medium before organoid placement (non-contact; medium only) and after organoid attachment (contact) across 16 channel electrodes (*n* = 16). Bar plots show electrode-wise impedance values and box plots summarize distributions (paired t-test; ^***^*p* < 0.001), demonstrating a consistent impedance reduction and low electrode-to-electrode variability after contact.

**Figure S26.** Geometrical parameters and FEA of the ultrathin MEA placed on a curved surface. a) Schematic illustration of the electrode width, hemisphere diameter under conformal attachment, and height of the MEA. b) Simulated displacement distribution applied to the MEA structure. c) Deformation under a thickness of ≈3.6 μm, shown from both side and front views.

**Reference**

[1]	a)B. Eisen, O. Binah, "Modeling Duchenne Muscular Dystrophy Cardiomyopathy with Patients' Induced Pluripotent Stem-Cell-Derived Cardiomyocytes," *Int J Mol Sci* **2023**, 24; b)V. Marini, F. Marino, F. Aliberti, et al., "Long-term culture of patient-derived cardiac organoids recapitulated Duchenne muscular dystrophy cardiomyopathy and disease progression," *Front Cell Dev Biol* **2022**, 10, 878311; c)C. Jung, A. S. Martins, E. Niggli, N. Shirokova, "Dystrophic cardiomyopathy: amplification of cellular damage by Ca2+ signalling and reactive oxygen species-generating pathways," *Cardiovasc Res* **2008**, 77, 766.

[2]	N. Shiba, X. Yang, M. Sato, et al., "Efficacy of exon-skipping therapy for DMD cardiomyopathy with mutations in actin binding domain 1," *Mol Ther Nucleic Acids* **2023**, 34, 102060.

[3]	B. Eisen, R. Ben Jehuda, A. J. Cuttitta, et al., "Electrophysiological abnormalities in induced pluripotent stem cell-derived cardiomyocytes generated from Duchenne muscular dystrophy patients," *J Cell Mol Med* **2019**, 23, 2125.

[4]	H. Yasutake, J. K. Lee, A. Hashimoto, et al., "Decreased YAP activity reduces proliferative ability in human induced pluripotent stem cell of duchenne muscular dystrophy derived cardiomyocytes," *Sci Rep* **2021**, 11, 10351.

[5]	Y. Song, J. C. Shryock, L. Belardinelli, "An increase of late sodium current induces delayed afterdepolarizations and sustained triggered activity in atrial myocytes," *Am J Physiol Heart Circ Physiol* **2008**, 294, H2031.

[6]	S. Wagner, N. Dybkova, E. C. Rasenack, et al., "Ca2+/calmodulin-dependent protein kinase II regulates cardiac Na+ channels," *J Clin Invest* **2006**, 116, 3127.

[7]	S. O. Marx, S. Reiken, Y. Hisamatsu, et al., "PKA phosphorylation dissociates FKBP12.6 from the calcium release channel (ryanodine receptor): defective regulation in failing hearts," *Cell* **2000**, 101, 365.

[8]	C. A. Remme, "Cardiac sodium channelopathy associated with SCN5A mutations: electrophysiological, molecular and genetic aspects," *J Physiol* **2013**, 591, 4099.

[9]	S. Verheule, T. Sato, T. t. Everett, et al., "Increased vulnerability to atrial fibrillation in transgenic mice with selective atrial fibrosis caused by overexpression of TGF-beta1," *Circ Res* **2004**, 94, 1458.

[10]	D. Dobrev, X. H. T. Wehrens, "Calcium-mediated cellular triggered activity in atrial fibrillation," *J Physiol* **2017**, 595, 4001.

[11]	R. Feiner, L. Engel, S. Fleischer, et al., "Engineered hybrid cardiac patches with multifunctional electronics for online monitoring and regulation of tissue function," *Nat Mater* **2016**, 15, 679.

[12]	L. Drakhlis, S. Biswanath, C. M. Farr, et al., "Human heart-forming organoids recapitulate early heart and foregut development," *Nat Biotechnol* **2021**, 39, 737.

[13]	a)P. Hofbauer, S. M. Jahnel, N. Papai, et al., "Cardioids reveal self-organizing principles of human cardiogenesis," *Cell* **2021**, 184, 3299; b)E. Giacomelli, V. Meraviglia, G. Campostrini, et al., "Human-iPSC-Derived Cardiac Stromal Cells Enhance Maturation in 3D Cardiac Microtissues and Reveal Non-cardiomyocyte Contributions to Heart Disease," *Cell Stem Cell* **2020**, 26, 862.

[14]	S. Shi, W. M. Hoogaars, D. J. de Gorter, et al., "BMP antagonists enhance myogenic differentiation and ameliorate the dystrophic phenotype in a DMD mouse model," *Neurobiol Dis* **2011**, 41, 353.

[15]	D. G. S. Wilson, A. Tinker, T. Iskratsch, "The role of the dystrophin glycoprotein complex in muscle cell mechanotransduction," *Commun Biol* **2022**, 5, 1022.

[16]	D. Fan, A. Takawale, J. Lee, Z. Kassiri, "Cardiac fibroblasts, fibrosis and extracellular matrix remodeling in heart disease," *Fibrogenesis Tissue Repair* **2012**, 5, 15.

[17]	A. R. Perestrelo, A. C. Silva, J. Oliver-De La Cruz, et al., "Multiscale Analysis of Extracellular Matrix Remodeling in the Failing Heart," *Circ Res* **2021**, 128, 24.

[18]	S. Zanotti, S. Saredi, A. Ruggieri, et al., "Altered extracellular matrix transcript expression and protein modulation in primary Duchenne muscular dystrophy myotubes," *Matrix Biol* **2007**, 26, 615.

[19]	U. o. Minnesota, "Development Timeline of Human Heart Embryology," *Atlas of Human Cardiac Anatomy,* University of Minniesota, **2024** <https://www.vhlab.umn.edu/atlas/congenital-defects-tutorial/development-timeline.shtml> (2024-05-22).
